# Procalcitonin and C-Reactive Protein Are Elevated at the Threshold of Cortical Electrocerebral Silence but Do Not Track Graded EEG Severity in Critically Ill Children: A Single-Centre Retrospective Cohort Study

**DOI:** 10.3390/brainsci16101012

**Published:** 2026-09-24

**Authors:** Robert Mîndreanu, Bogdan Florea, Denisa Ștefănescu, Orlanda Moldovan, Sorin Claudiu Man, Gabriela Adriana Filip, Camelia Alexandra Coadă, Simona Clichici

**Affiliations:** 1 Division of Physiology, Department of Morpho-functional sciences, Iuliu Hațieganu University of Medicine and Pharmacy, 400132 Cluj-Napoca, Romania; robert.flor.mindreanu@elearn.umfcluj.ro (R.M.);; 2 Centre for Epilepsy and EEG Monitoring, 400427 Cluj-Napoca, Romania; 3 Department of Neurology, Faculty of Medicine, Iuliu Hațieganu University of Medicine and Pharmacy, 400132 Cluj-Napoca, Romania; 4 Third Pediatric Discipline, Mother and Child Department, Faculty of Medicine, Iuliu Hațieganu University of Medicine and Pharmacy, 400132 Cluj-Napoca, Romania; 5 Third Pediatric Clinic, Emergency Clinical Hospital for Children, 400132 Cluj-Napoca, Romania; 6 Department of Anatomy and Embryology, Iuliu Hațieganu University of Medicine and Pharmacy, 400132 Cluj-Napoca, Romania

**Keywords:** coma, EEG, clinical neurology, inflammation, paediatric neurology

## Abstract

Background: The relationship between routine inflammatory biomarkers and EEG severity in critically ill children, evaluated using visual (Synek) grading and spectral qEEG, remains unclear. The aim of our study was to examine the associations between procalcitonin (PCT), C-reactive protein (CRP) and EEG severity. Methods: Single-centre retrospective cohort of 97 critically ill children were analysed (PICU, Romania; November 2022–February 2026), each contributing one EEG with an inflammatory biomarker measured within ≤24 h. The primary analysis was the Spearman correlation of PCT and CRP with Synek grade, supported by comparisons of cortical electrocerebral silence with preserved cortical activity (Synek 1–4). The secondary analysis comprised five age-adjusted partial Spearman correlations between biomarkers and spectral qEEG metrics in the spectral cohort (*n* = 71), BH-corrected within family. Results: PCT and CRP were markedly higher in cortical silence than in preserved activity (Mann–Whitney *p*-value = 9.6 × 10^−6^ and 4.1 × 10^−6^; adjusted OR ≈ 2 per ln-unit). Both markers showed a positive rank association across Synek grades (PCT ρ = +0.49; CRP ρ = +0.41; both BH-adjusted *p*-value < 10^−4^), driven by the Synek-5 step: within grades 1–4 neither was graded (PCT ρ = +0.19; CRP ρ = +0.08; both *p*-value > 0.1). None of the five secondary correlations suggested a moderate-to-strong association (ρ −0.14 to +0.08; all BH-adjusted *p*-value ≥ 0.668). Conclusions: PCT and CRP were elevated at the threshold of cortical electrocerebral silence but did not provide a graded severity index within Synek grades 1–4, nor did they associate with spectral qEEG, including in the full 10–20 subset. The spectral analysis was underpowered for weak effects and excluded the Synek-5 subset where the primary signal was localised.

## 1. Introduction

Acute brain dysfunction, ranging from encephalopathy and impaired consciousness to electrographic seizures, is common among critically ill children and carries substantial prognostic weight for survival and long-term neurodevelopmental outcome [1,2]. Systemic inflammatory states are increasingly recognised as important contributors to this dysfunction. In sepsis, severe infection and hypoxic–ischaemic injury, circulating pro-inflammatory mediators may affect the brain through neuroinflammatory activation, blood–brain barrier disruption, microcirculatory failure, and cytokine-mediated neuronal and synaptic dysfunction, a constellation clinically recognised as sepsis-associated encephalopathy [3,4].

Electroencephalography (EEG) is the principal bedside tool for detecting and grading this dysfunction in the intensive care unit [5]. Visual severity scales, of which the Synek classification is among the most widely used in coma and encephalopathy, provide an ordinal grading of background organisation that correlates with depth of encephalopathy and with outcome [5,6]. Quantitative EEG (qEEG) extends this assessment with spectral measures: absolute and relative band power and composite slowing indices quantify background slowing on a continuous scale and have been applied in adult and paediatric critical care, where they may track injury severity and prognosis [7,8,9].

Routine inflammatory biomarkers (C-reactive protein (CRP) and procalcitonin (PCT) foremost) are widely used in critically ill children as part of standard care [10,11]. To our knowledge, no published study has directly examined the association between routine systemic inflammatory biomarkers and quantitative spectral qEEG parameters in critically ill children. An important distinction must be noted at the outset: peripheral systemic inflammatory signals and central neuroinflammatory processes are biologically coupled through blood–brain barrier-mediated neuroimmune pathways, but they remain compartmentally and mechanistically non-identical [12].

We therefore examined the association between routine systemic inflammatory biomarker burden, indexed by PCT and CRP, and EEG severity at two complementary levels (Synek grading and quantitative spectral qEEG) in a single-centre cohort of critically ill children, with adjustment for age. Given the absence of prior data directly addressing this question, we approached it as an exploratory analysis. We hypothesised that higher inflammatory biomarker burden would be associated with greater EEG severity at both levels, while recognising that systemic biomarkers may also reflect global critical illness severity rather than brain-specific inflammatory mechanisms.

## 2. Materials and Methods

### 2.1. Study Design and Setting

This is a retrospective, observational, single-centre cohort study conducted in the paediatric intensive care unit (PICU) of Children’s Emergency Clinical Hospital Cluj-Napoca, Romania, between November 2022 and February 2026. Reporting follows the STROBE statement for observational studies [13]. The STROBE checklist is available as Supplementary Materials S1.

### 2.2. Participants

Consecutive critically ill children admitted to the PICU during the study period who underwent at least one clinically indicated EEG were screened. Inclusion criteria: (a) age < 18 years at admission; (b) at least one clinically indicated EEG; (c) at least one routine inflammatory biomarker (CRP or PCT) measured within ≤24 h, before or after the EEG recording. Laboratory values outside the 24 h window were not used.

Exclusion criteria for the spectral analysis: Cortical electrocerebral silence was retained for Synek-based analyses but excluded from quantitative spectral analyses on methodological grounds (Welch spectral estimation on an essentially flat trace reflects instrumental noise rather than cortical activity). Patients of all paediatric ages were retained to preserve the heterogeneity of a real-world PICU population; montage heterogeneity was addressed in a sensitivity analysis restricted to the full 10–20 subset.

### 2.3. Cohort Stratification

The cohort was partitioned into three subsets: the spectral cohort (*n* = 71; usable qEEG), comprising 19 recordings from children aged 0–7 months and 52 from children aged ≥ 8 months, and the cortical silence cohort (*n* = 26), included in Synek-based analyses but excluded from spectral analyses.

### 2.4. Clinical Data

Demographics, weight, length of PICU stay and in-hospital mortality were extracted from the electronic clinical record. Each patient was assigned one of three diagnostic categories: epilepsy (*n* = 23 in the spectral cohort); inflammatory brain injury (sepsis + meningoencephalitis, *n* = 33); hypoxic–ischaemic injury (post-cardiac arrest + post-hypoxic encephalopathy, *n* = 13). Trauma (*n* = 2 in the spectral cohort; *n* = 8 overall, 6 with cortical silence) was excluded from diagnostic subgroup analyses on sample-size grounds but retained in all other analyses. Validated paediatric illness severity scores were not reliably available in the retrospective dataset.

### 2.5. Inflammatory Biomarkers and Laboratory Variables

Routine peripheral biomarkers temporally closest to the EEG and within ≤24 h were extracted: CRP, PCT, white blood cell, neutrophil and lymphocyte counts, platelets, haemoglobin, albumin, AST, ALT and creatinine, with neutrophil-to-lymphocyte and platelet-to-lymphocyte ratios derived. Lactate, fibrinogen and ferritin were available in a minority of records (lactate 7/97, including one child with cortical silence; fibrinogen 26/97; ferritin 16/97) and were not analysed. Vasopressor support and mechanical ventilation status were not collected, as the data collection protocol was designed to examine routine inflammatory biomarkers in relation to EEG.

### 2.6. EEG Acquisition and Sedation

EEG was recorded on a NATUS clinical system (Natus Medical Incorporated, Middleton, WI, USA) exported to European Data Format (EDF). Per institutional protocol, children aged ≥ 8 months were recorded using the standard international 10–20 montage and those aged 0–7 months using an intermediate 11-electrode infant montage; the institutional protocol required continuous sedative infusions to be withdrawn ≥ 30 min before EEG initiation, but adherence to this requirement, the agents used and the timing of the last dose could not be reliably extracted from the retrospective record.

### 2.7. EEG Visual Review and Synek Grading

All EEGs were reviewed by a senior neurophysiologist, blinded to biomarker values, and graded according to the Synek classification [6]: grade 1 (mostly alpha with reactivity), 2 (predominantly theta), 3 (predominantly delta), 4 (low-voltage delta or burst-suppression), 5 (cortical electrocerebral silence). Because the Synek scale was developed in adults, though subsequently applied in paediatric and young adult critical populations, where background grades tracked clinical severity [14], and comparable five-grade background scales ranging from normal to isoelectric have shown prognostic value in children after cardiac arrest [15], its frequency criteria were applied relative to the background activity expected for age, as in routine paediatric EEG interpretation. Grade 1 therefore denoted a reactive background with age-appropriate dominant frequencies (in infants, without a posterior alpha rhythm), and grades 2 and 3 predominantly theta and delta slowing beyond that expected for age. Grades 1–4 were thus used as ordinal descriptors rather than as a validated paediatric scale, whereas grade 5 does not depend on the maturation of background rhythms. Grade 5 was assigned at the recording level when no identifiable cerebral background activity was present on visual inspection, after exclusion of technical artefact and confirmation that the recording was technically interpretable. A second neurophysiologist, blinded to the first rating and to all clinical and laboratory data, independently graded every included recording (*n* = 97) to assess inter-rater reliability.

### 2.8. Quantitative EEG (qEEG) Processing

EDF files were processed in MNE-Python 1.12.0 [16] and spectral analysis performed in MATLAB R2024b (The MathWorks, Inc., Natick, MA, USA) with EEGLAB 2026.0.0 [17]. Recordings were bandpass-filtered between 0.5 and 45 Hz with a 50 Hz notch and ocular/muscular artefacts were removed by independent component analysis [18]. Artefact-free awake-or-baseline interictal segments were selected manually by a rater blinded to clinical group, using a bipolar double-banana display for visual inspection only. The selected segments were cut from the referential recordings (up to 18 channels in the 10–20 montage and fewer in the 11-electrode infant montage, after exclusion of channels judged unusable) and concatenated. In EEGLAB, signals were re-referenced to the common average of the available channels, and the power spectral density of each channel was estimated by Welch’s method (2 s Hann windows, 50% overlap, 4 s FFT length with zero-padding, 0.25 Hz bins); windows spanning junctions between concatenated segments were not excluded. Because each junction can affect only the few Hann-tapered windows that overlap it, and because the number of junctions is unrelated to biomarker values, any resulting broadband artefact is expected to be small and non-differential between patients. Absolute power (µV^2^) was obtained by summing the spectral density over δ (1–4), θ (4–8), α (8–13) and β (13–30 Hz), and total power over 1–30 Hz, for each channel and then averaged across channels; relative power was computed per channel before averaging. The delta–alpha ratio (DAR) and delta–theta–alpha–beta ratio (DTABR) were derived from the channel-averaged absolute band powers, and the brain symmetry index (BSI) was computed as the mean, across available homologous electrode pairs, of |P_left − P_right|/(P_left + P_right), where P is the total power of each electrode. Peak alpha frequency, taken as the maximum of the channel-averaged spectrum between 8 and 13 Hz, was non-identifiable in 60 of 71 recordings (84.5%), in which this maximum fell on a band edge, and was retained only as a descriptive flag. Identifiability did not vary with Synek grade (11–22% across grades 1–4), suggesting a methodological rather than severity-related cause: a fixed adult alpha band applied to a cohort in which 41 of 71 children were younger than 3 years, recorded in the PICU rather than in relaxed eyes-closed wakefulness. Only the first clinically obtained EEG per patient was used.

### 2.9. Statistical Analysis

Analyses were organised as primary, secondary and exploratory, with Benjamini–Hochberg (BH) false discovery rate correction [19] applied within each family of tests. No prospective registration was undertaken. The analysis plan was defined after data collection; the primary and secondary tests were specified before their results were inspected, and all other analyses were supporting, exploratory or post hoc (Supplementary Materials S2, Section S2.1). By design, Synek grade 5 (*n* = 26) was eligible for Synek-based analyses but excluded from spectral analyses. Tests were two-tailed at α = 0.05. Non-parametric methods were used because most laboratory and spectral variables were right-skewed and included extreme but clinically genuine values; distributional assumptions were not formally tested. The test used for each type of comparison is listed in Supplementary Materials S2 (Table S2.5). All analyses were performed in Stata 19.5 BE (StataCorp LLC, College Station, TX, USA). Missing laboratory values reflected clinical ordering rather than study procedures: CRP was part of routine laboratory panels, whereas PCT was requested at the clinician’s discretion. PCT was missing in 13 of 71 children with preserved cortical activity and in 2 of 26 with cortical silence. Analyses were complete-case, without imputation.

The two primary tests were Spearman correlations of PCT and CRP with Synek grade across the combined cohort (*n* = 97; effective *n* PCT 82, CRP 93), with BH correction across both. Because grade 5 is a qualitatively distinct state rather than a further step of slowing, a positive correlation across the full scale was not taken as evidence of a graded relationship, and its source was examined in supporting analyses: Mann–Whitney U tests and logistic regression on ln-transformed markers compared cortical electrocerebral silence (Synek 5) with preserved cortical activity (Synek 1–4), and Spearman correlations within grades 1–4 tested for a graded association below the silence threshold.

Secondary analyses comprised five age-adjusted partial Spearman correlations in the spectral cohort (*n* = 71; effective *n* 57–69; implementation in Supplementary Materials S2, Section S2.3): PCT and CRP each against DTABR and absolute delta power, and a composite inflammatory score against total spectral power. The composite was the unweighted mean of robust z-scores of ln-transformed PCT and CRP. Equal weights were used because no external criterion exists for weighting the two markers (Supplementary Materials S2, Section S2.2). No a priori sample size calculation was performed, as the sample was fixed by the retrospective design. After the analyses, we calculated the smallest correlation detectable with 80% power (two-sided α = 0.05) at each available sample size (Supplementary Materials S2, Section S2.8). Confidence intervals for the partial correlations were obtained by Fisher z transformation with standard error 1/√(*n* − 4), which accounts for the single covariate (Supplementary Materials S2, Section S2.3). AST and platelet count were examined against Synek grade post hoc, because they differed between the cohorts; these analyses are hypothesis-generating.

Exploratory, robustness and sensitivity analyses are specified in full in Supplementary Materials S2: a laboratory × qEEG grid (13 markers × 12 metrics), qEEG metrics against Synek grade, diagnostic subgroup correlations, adjustment for age and creatinine, outlier and artefact trimming, and replication in the montage-homogeneous standard 10–20 subset (*n* = 52). Because no validated paediatric severity score was available, residual confounding by global illness severity remains the principal limitation.

## 3. Results

### 3.1. Study Population

Of 103 critically ill children meeting the eligibility criteria, six were excluded on EEG or data-availability grounds, leaving 97 in the principal analyses (Figure 1). Of these, 71 contributed an artefact-free qEEG suitable for spectral analysis (Synek 1–4; spectral cohort) and 26 met electrographic criteria for cortical silence (Synek 5). Within the spectral cohort, admission diagnoses were inflammatory (*n* = 33), epileptic (*n* = 23) and hypoxic–ischaemic (*n* = 13); two trauma patients were retained but excluded from diagnostic subgroup analyses. The Synek grade distribution within the spectral cohort was 9, 18, 24 and 20 at grades 1–4, respectively.

### 3.2. Cohort Characteristics

Baseline characteristics are summarised in Table 1. Compared with the spectral cohort, children with cortical silence were older (median 82.5 vs. 24.0 months; *p*-value = 0.010), had a shorter length of stay and substantially higher in-hospital mortality (42.3% vs. 7.0%; Fisher exact *p*-value < 0.001), and showed higher systemic inflammatory and organ-dysfunction markers (CRP, PCT, AST) with lower platelets and albumin, consistent with greater overall illness severity.

### 3.3. Inflammatory Markers and the Threshold of Cortical Electrocerebral Silence (Primary Analysis)

PCT and CRP were markedly higher in recordings with cortical electrocerebral silence (Synek 5) than in those with preserved cortical activity (Mann–Whitney U *p*-value = 9.6 × 10^−6^ for PCT; *p*-value = 4.1 × 10^−6^ for CRP; Table 1 and Table 2). Across the combined cohort (Synek 1–5), both markers showed a positive rank association with Synek grade: PCT ρ = +0.49 (95% CI +0.31 to +0.64; raw *p*-value = 3.8 × 10^−6^; BH-adjusted *p*-value = 7.5 × 10^−6^; *n* = 82) and CRP ρ = +0.41 (95% CI +0.23 to +0.57; raw *p*-value = 5.1 × 10^−5^; BH-adjusted *p*-value = 5.1 × 10^−5^; *n* = 93). Both survived BH correction.

This rank correlation was generated by the discontinuity at Synek 5 rather than by a graded relationship through grades 1–4. Medians across Synek 1, 2, 3, 4 were 0.24, 0.18, 0.25, 0.39 ng/mL for PCT and 1.22, 0.38, 0.60, 0.64 mg/dL for CRP—overlapping distributions with no monotonic trend; at Synek 5 the medians were 3.22 ng/mL and 12.34 mg/dL, an approximately 8-fold and 19-fold elevation over the grade-4 central tendency (Table 2; Figure 2). Within Synek 1–4 alone, neither marker showed a graded ordinal association (PCT ρ = +0.19, *p*-value = 0.15, *n* = 58; CRP ρ = +0.08, *p*-value = 0.50, *n* = 69). We therefore interpret the primary association as a cortical silence-driven contrast, not evidence of a graded inflammatory markers–EEG relationship within Synek 1–4. Cortical silence was most frequent after trauma (6/8) and hypoxic–ischaemic injury (12/25) and least frequent in inflammatory illness (6/39) and epilepsy (2/25). Within each systemic aetiology, CRP and PCT were higher in cortical silence than in preserved activity, whereas the two children with epilepsy and cortical silence had values similar to those of other children with epilepsy (Supplementary Table S2.4).

### 3.4. Inflammatory Markers and Quantitative Spectral qEEG (Secondary Analysis)

No moderate-to-strong association between inflammatory markers and spectral qEEG was detected in the five secondary partial Spearman correlations: PCT × DTABR (ρ = +0.08; 95% CI −0.18 to +0.34; raw *p*-value = 0.53; *n* = 58); PCT × absolute delta (ρ = −0.10; 95% CI −0.36 to +0.16; *p*-value = 0.44; *n* = 58); CRP × DTABR (ρ = −0.02; 95% CI −0.26 to +0.22; *p*-value = 0.85; *n* = 69); CRP × absolute delta (ρ = −0.11; 95% CI −0.34 to +0.13; *p*-value = 0.37; *n* = 69); composite inflammatory score × total spectral power (ρ = −0.14; 95% CI −0.39 to +0.13; *p*-value = 0.31; *n* = 57; permissive composite *n* = 70 gave ρ = −0.11, *p*-value = 0.37). All point estimates were small (|ρ| ≤ 0.14) and all BH-adjusted *p*--values were ≥0.668. The 95% confidence intervals excluded moderate-to-strong associations for the majority of tests but remained compatible with weak true effects in either direction.

Because 19 children aged 0–7 months were recorded with an intermediate 11-electrode montage, we repeated the five secondary correlations in the montage-homogeneous full 10–20 subset (age ≥ 8 months; *n* = 52; effective *n* 41–50). The null pattern was unchanged: all |ρ| ≤ 0.16, all BH-adjusted *p*-value ≥ 0.807, with no change in direction relative to the full spectral cohort (Supplementary Materials S2). The distribution of spectral qEEG metrics across Synek grades 1–4 is shown in Figure 3.

### 3.5. Post Hoc Analyses: Systemic Severity Markers and Synek Grade

Two organ-dysfunction markers correlated with Synek grade across the combined cohort: AST ρ = +0.34 (95% CI +0.14 to +0.51; raw *p*-value = 1.1 × 10^−3^; BH-adjusted *p*-value = 2.0 × 10^−3^; *n* = 90) and platelet count ρ = −0.32 (95% CI −0.49 to −0.12; raw *p*-value = 2.0 × 10^−3^; BH-adjusted *p*-value = 2.0 × 10^−3^; *n* = 91). Because these markers were selected after inspection of the cohort differences, the associations are post hoc and hypothesis-generating, and they are reported as markers of global severity rather than inflammation-specific findings.

### 3.6. Exploratory Analyses

No exploratory or sensitivity analysis produced a result surviving BH correction. In the laboratory × qEEG grid (156 age-adjusted partial correlations), coefficients ranged from −0.42 to +0.35 (smallest BH-adjusted *p*-value = 0.077); the inflammatory-marker columns were uniformly weak, the strongest overall signal being AST × relative theta (ρ = −0.42, raw *p*-value < 0.001) (Figure 4). No qEEG metric showed a monotonic or quadratic association with Synek grade surviving correction. Of 468 diagnostic subgroup correlations, twenty were nominally significant and none survived global BH-FDR correction; the strongest, AST versus relative theta in the epilepsy subgroup, is shown in Figure 5. The secondary null was reproduced in all four sensitivity analyses (relative beta ≤ 25%, 3 × IQR outlier trimming, the montage-homogeneous 10–20 subset [*n* = 52] and exclusion of neonates aged < 1 month), with no BH-significant pair in any (Supplementary Materials S2). These analyses are hypothesis-generating only.

### 3.7. Robustness, Sensitivity and Inter-Rater Reliability

The primary inflammation–Synek association persisted after adjustment for age and renal function. Logistic regression for the cortical silence contrast gave adjusted odds ratios of ≈2 per natural-log unit for both markers (PCT: unadjusted OR 1.93, 95% CI 1.40–2.66; age- and creatinine-adjusted OR 2.00, 1.40–2.85; CRP: 2.26, 1.55–3.30 and 2.19, 1.48–3.24). Creatinine was not independently associated with cortical silence in either adjusted model (*p*-value = 0.38 and *p*-value = 0.95). With 24 events, the adjusted models had about eight events per parameter; a model additionally including diagnostic category, with about four events per parameter, is reported in Supplementary Materials S2 (Section S2.6) but not interpreted. No model was adjusted for validated global illness severity.

Inter-rater reliability of Synek grading against a second reader blinded to the first rating and to clinical data (*n* = 97) showed 89.7% observed agreement, Cohen κ = 0.87 (unweighted) and quadratic-weighted κ = 0.97; all discordances were between adjacent grades, and agreement on the cortical silence-versus-1–4 dichotomy was perfect (κ = 1.00).

### 3.8. Outcome

In-hospital mortality was concentrated in the cortical silence cohort (11/26, 42.3%) versus the spectral cohort (5/71, 7.0%). In univariable logistic regression, higher Synek grade (OR 2.33, 95% CI 1.32–4.12, *p*-value = 0.004) and higher ln-PCT (OR 1.51, 1.12–2.04, *p*-value = 0.008) were associated with mortality, whereas ln-CRP was not (OR 1.33, 0.95–1.87, *p*-value = 0.10). With events few and concentrated in Synek 5, these unadjusted associations largely recapitulate the cortical silence contrast rather than an independent prognostic effect.

## 4. Discussion

In this cohort of critically ill children, routine inflammatory markers carried information about EEG state at only one, categorical, level: PCT and CRP were strongly elevated in children whose EEG showed cortical electrocerebral silence, but were essentially uninformative about the degree of dysfunction in EEGs that retained cortical activity. The full-range rank correlation, though statistically clear, was produced entirely by the step at the silence threshold rather than by any continuous gradient through the preserved-activity grades. Cortical silence was the one grading decision on which two blinded readers agreed perfectly (κ = 1.00), so the contrast is unlikely to reflect misclassification. The most likely alternative explanation is global illness severity. Children with cortical silence also had higher in-hospital mortality (42.3% vs. 7.0%) and worse organ dysfunction markers (Table 1), and CRP and PCT rise with systemic injury of many causes, not only infection; their elevation may therefore reflect overall illness severity rather than the EEG state itself. Most children with cortical silence had hypoxic–ischaemic or traumatic rather than infectious illness, and the marker elevation was present within each systemic aetiology (Supplementary Table S2.4). Because cortical silence is itself partly a consequence of that severity, the two cannot be separated in this retrospective design.

At the finer, spectral level no inflammation–EEG relationship emerged, the confidence intervals rendering a moderate-to-strong effect unlikely while leaving weak effects open. This null becomes more interpretable given the nature of the spectral cohort: these were predominantly abnormal recordings, an identifiable posterior alpha rhythm was absent in most, and the spectral slowing indices themselves did not rise monotonically with visual severity, peaking and then falling as total power collapsed at the most suppressed preserved grade. The same null pattern was found in the montage-homogeneous 10–20 subset, so it is unlikely to reflect the combination of montages with different electrode coverage. This decline most likely reflects a change in phenotype rather than an improvement. Grade 4, defined by low-voltage delta or burst-suppression, is characterised by loss of amplitude, whereas grades 2–3 are characterised by slowing: median total power fell from 138.6 µV^2^ at grade 3 to 39.8 µV^2^ at grade 4. Ratio indices such as DAR and DTABR are insensitive to a global loss of amplitude and become unstable as band power approaches the noise floor, so they capture slowing but not suppression. Slowing and suppression are therefore distinct electrophysiological dimensions, and a continuous inflammatory signal cannot be expected to track spectral indices that do not behave monotonically across them.

These observations sit alongside a qEEG literature in which spectral slowing indexes cerebral dysfunction that follows a defined cerebral insult (ischaemic, metabolic or neurotoxic) rather than the systemic inflammatory state as such: the delta/alpha ratio in acute ischaemic stroke [8], delayed cerebral ischaemia after subarachnoid haemorrhage [7], encephalopathy grading in paediatric acute liver failure [9], and background grading in neurotoxicity-related delirium [14]. Where qEEG has been linked to inflammation, the informative signals have been central rather than peripheral: in a prospective PICU cohort of sepsis and meningoencephalitis, quantitative but not routine visual EEG correlated with biomarkers of brain injury [20], and qEEG indices tracked inflammatory and metabolic factors in Parkinson’s disease dementia [21]. The sepsis-associated encephalopathy (SAE) literature is the most directly relevant comparator, since inflammatory diagnoses formed the largest aetiological group here: in SAE, EEG shows a graded but non-specific abnormality while blood biomarkers of brain injury have limited diagnostic value [4,22].

Our data cannot distinguish between two explanations: a dissociation between systemic and central inflammation, and confounding by global illness severity. CRP and PCT are coupled to, but not identical with central neuroinflammation [12], and diverge precisely where the cerebral insult is not primarily inflammatory: hypoxic–ischaemic and post-arrest injury, both well represented here. Cortical silence, by contrast, is reached through several converging pathways, most of which accompany the advanced systemic illness that also drives CRP and PCT upward; it is at this extreme that systemic and cerebral injury most reliably coincide. The signal persisted after adjustment for age, renal function and diagnostic category but could not be separated from overall illness severity. We therefore read the inflammation–silence link as a non-specific marker of the advanced critical-illness state rather than an inflammation-specific or causal effect on cortical function.

### Limitations

This was a retrospective single-centre study, and unmeasured confounding cannot be excluded. Most importantly, no validated paediatric severity score was available, so the inflammation–silence association could not be separated from global illness severity. Surrogate severity indicators were also limited: lactate was measured in too few patients to be used, and vasopressor support and mechanical ventilation were not recorded. The primary signal is localised to the contrast between cortical silence and preserved activity, not to a graded gradient across Synek 1–4; the Spearman ρ across the full range should not be read as evidence of a continuous gradient. The secondary spectral analysis was underpowered for weak effects (ρ ≈ 0.33–0.37 required for 80% power at the available effective *n* of 57–69), so weak associations cannot be excluded, although the reported CIs render moderate-to-strong associations unlikely.

Measurement limitations apply. Sedation is a potential confounder. The protocol-mandated washout of ≥30 min is shorter than the elimination half-life of commonly used sedatives, adherence to it was not verified, and the agents used and the interval between the last dose and the EEG were not available; residual sedative effects on the EEG background and spectral metrics therefore cannot be excluded. The relative-beta ≤ 25% sensitivity analysis provides only indirect control and does not capture agents that do not increase beta activity. Biomarkers were measured within ±24 h either side of the EEG, so directionality cannot be inferred, and a single value represents one point in a dynamic process. Exact sampling times were not recorded, so the actual interval could not be quantified or narrowed. CRP and PCT index peripheral inflammatory burden rather than central neuroinflammation; markers of central injury (NfL, GFAP, S100B, CSF cytokines) were unavailable. Children aged 0–7 months were recorded with an 11-electrode infant montage; because average-reference estimates depend on electrode coverage, spectral values are not interchangeable between montages at the individual level, which is why the secondary analyses were repeated in the montage-homogeneous 10–20 subset. Peak alpha frequency was non-extractable in 84.5% of recordings. AST and platelet count were selected after inspection of the cohort differences, and the BH correction applied to these tests does not account for that selection step.

The Synek scale was developed in adults and its age-referenced application in children relied on the readers’ judgement rather than on validated paediatric criteria. Boundaries between adjacent grades 1–4 may therefore be less reliable in young children, and the within-grades-1–4 analyses should be read with this caveat. In critically ill children, EEG features based on predominant frequency are less reproducible between readers than those based on continuity and suppression [23]; consistent with this, all discordances between our two readers were between adjacent grades, and agreement on the cortical silence dichotomy was perfect (κ = 1.00). Infants were retained to reflect the real-world PICU population; only 1 of the 26 children with cortical silence was younger than 8 months. The primary finding rests on a dichotomy defined identically at all ages and is less affected by it.

Functional connectivity and cross-frequency coupling describe interactions between frequency components that band power does not capture, and both have been computed from clinical EEG in comatose children [24]. In a non-systematic PubMed search, we found only small or exploratory studies relating these measures to inflammatory markers, mainly in adults with delirium [25,26], and none in critically ill children; their relation to systemic inflammation therefore remains undetermined. In children, these measures are feasible and developmentally sensitive: phase–amplitude coupling stratified encephalopathy severity in neonatal hypoxic–ischaemic encephalopathy [27], phase–phase and phase–amplitude interactions have been measured in infant cortical networks, including after prenatal antidepressant exposure [28], coupling between slow-wave and temporal theta activity changes systematically with gestational age in preterm neonates [29], and cross-frequency coupling has been applied to paediatric epilepsy in magnetoencephalographic recordings in childhood absence epilepsy [30]. More recent paediatric work extends this to scalp EEG and to clinically relevant states: theta–gamma and delta–beta coupling measured during infancy predicted language outcomes one year later [31], delta–alpha coupling is stronger during sevoflurane anaesthesia than during emergence in young children [32], and delta–high-frequency coupling has been used as a spatial biomarker in paediatric drug-resistant epilepsy, albeit on intracranial recordings [33]. These measures are therefore sensitive to both age and sedation, the two factors we could not control here, and would require explicit handling before they could be related to systemic inflammation in a critically ill paediatric cohort; they nevertheless remain a plausible next step for relating systemic inflammation to cortical network function.

## 5. Conclusions

In critically ill children, systemic inflammatory markers were associated with EEG severity through a threshold rather than a graded pattern. Procalcitonin and C-reactive protein were markedly elevated in cortical silence, but did not track background severity within the gradable range and showed no moderate-to-strong association with quantitative EEG spectral content. Because this association could not be separated from overall illness severity, it is best interpreted as a non-specific marker of advanced critical illness. Inflammatory markers cannot grade, reconstruct or exclude encephalopathy, for which direct neurophysiological evaluation remains indispensable.

## Figures and Tables

**Figure 1:**
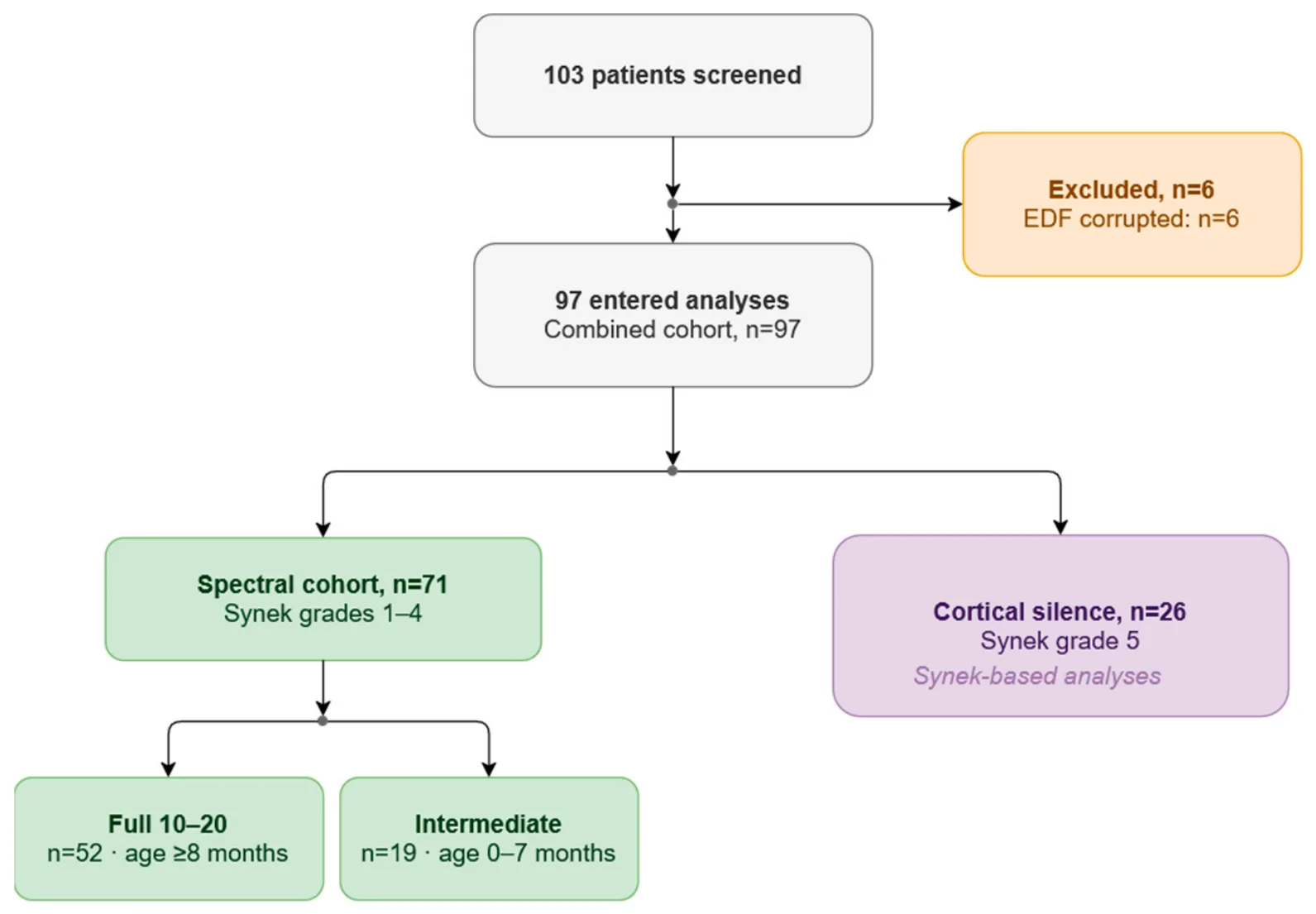
Study flowchart. Of 103 enrolled children, six were excluded on EEG or data-quality grounds; 97 entered analyses. The combined cohort (*n* = 97) was used for the primary analysis; the spectral subcohort (Synek 1–4, *n* = 71) for quantitative analyses; the cortical silence subcohort (Synek 5, *n* = 26) for Synek-based analyses only.

**Figure 2:**
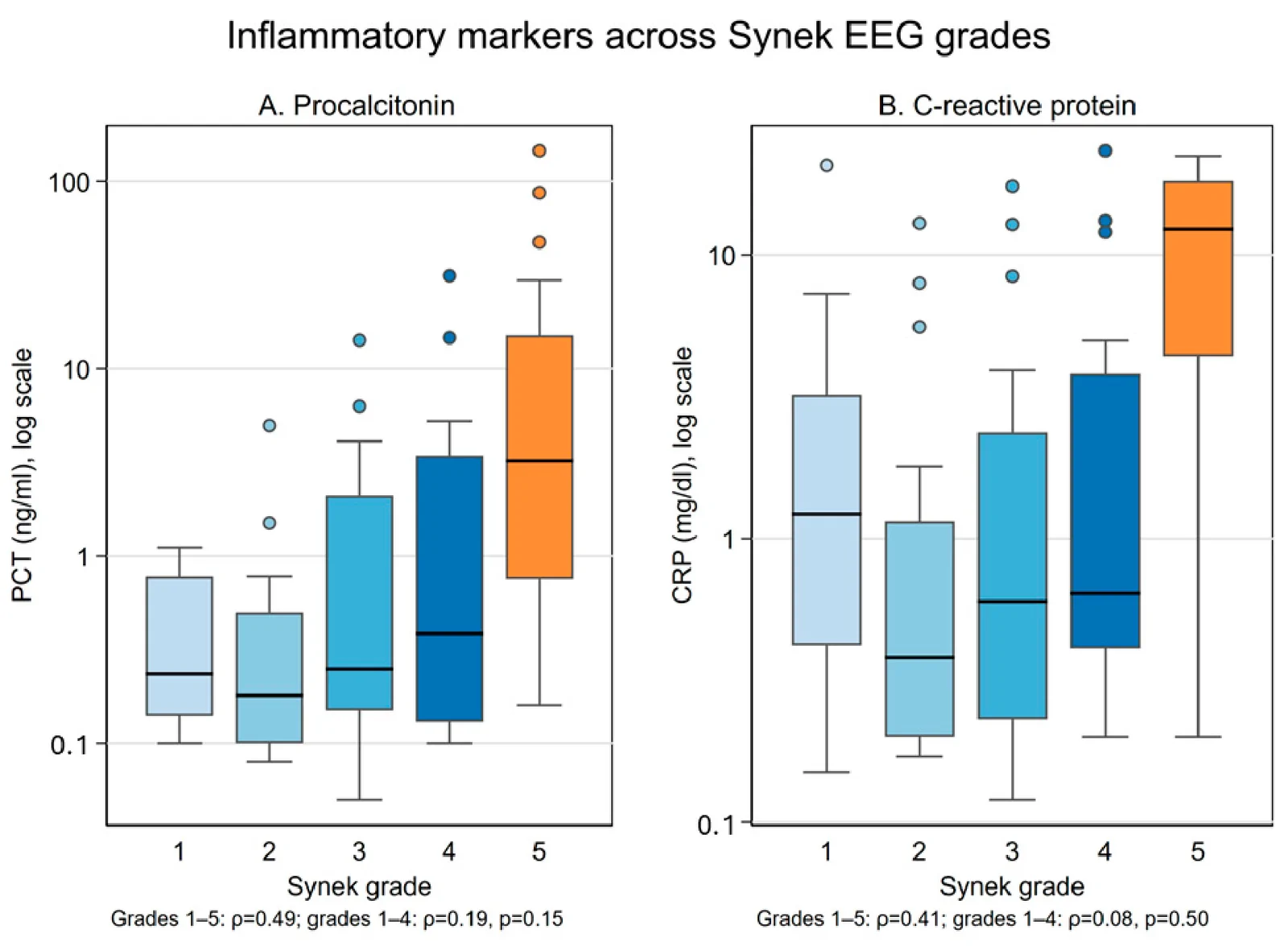
The rank correlation across all five grades (PCT ρ = +0.49, CRP ρ = +0.41; both BH-adjusted *p*-value < 0.001) reflects the step at grade 5; within grades 1–4, no graded association was detected (PCT ρ = +0.19, *p*-value = 0.15; CRP ρ = +0.08, *p*-value = 0.50).

**Figure 3:**
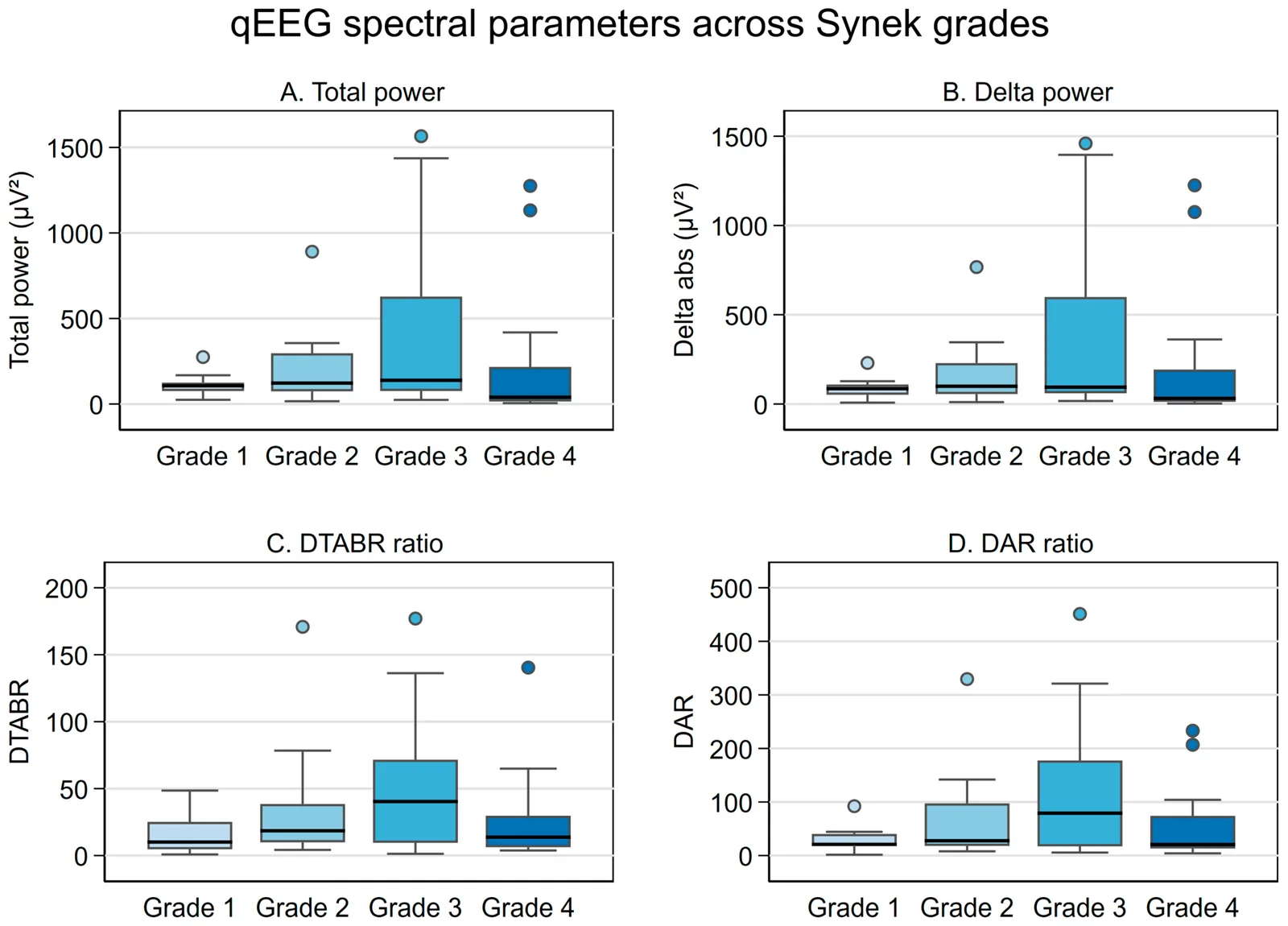
Quantitative spectral qEEG parameters across Synek grades 1–4 (spectral cohort, *n* = 71). Box plots show median and IQR; whiskers extend to 1.5 × IQR. Panels show total spectral power (µV^2^), absolute delta power (µV^2^), DTABR and DAR. Median DTABR across grades 1, 2, 3, 4: 10.1, 18.5, 40.3, 13.8; median DAR: 20.8, 27.7, 79.1, 20.5. The pattern is non-monotonic: values peak at grade 3 and decline at grade 4 as total spectral power collapses rather than continuing to slow. No qEEG metric showed a monotonic association with Synek grade surviving BH correction (strongest signal: absolute alpha ρ = −0.24, BH-adjusted *p*-value = 0.34). DTABR, (delta + theta)/(alpha + beta) ratio; DAR, delta/alpha ratio.

**Figure 4:**
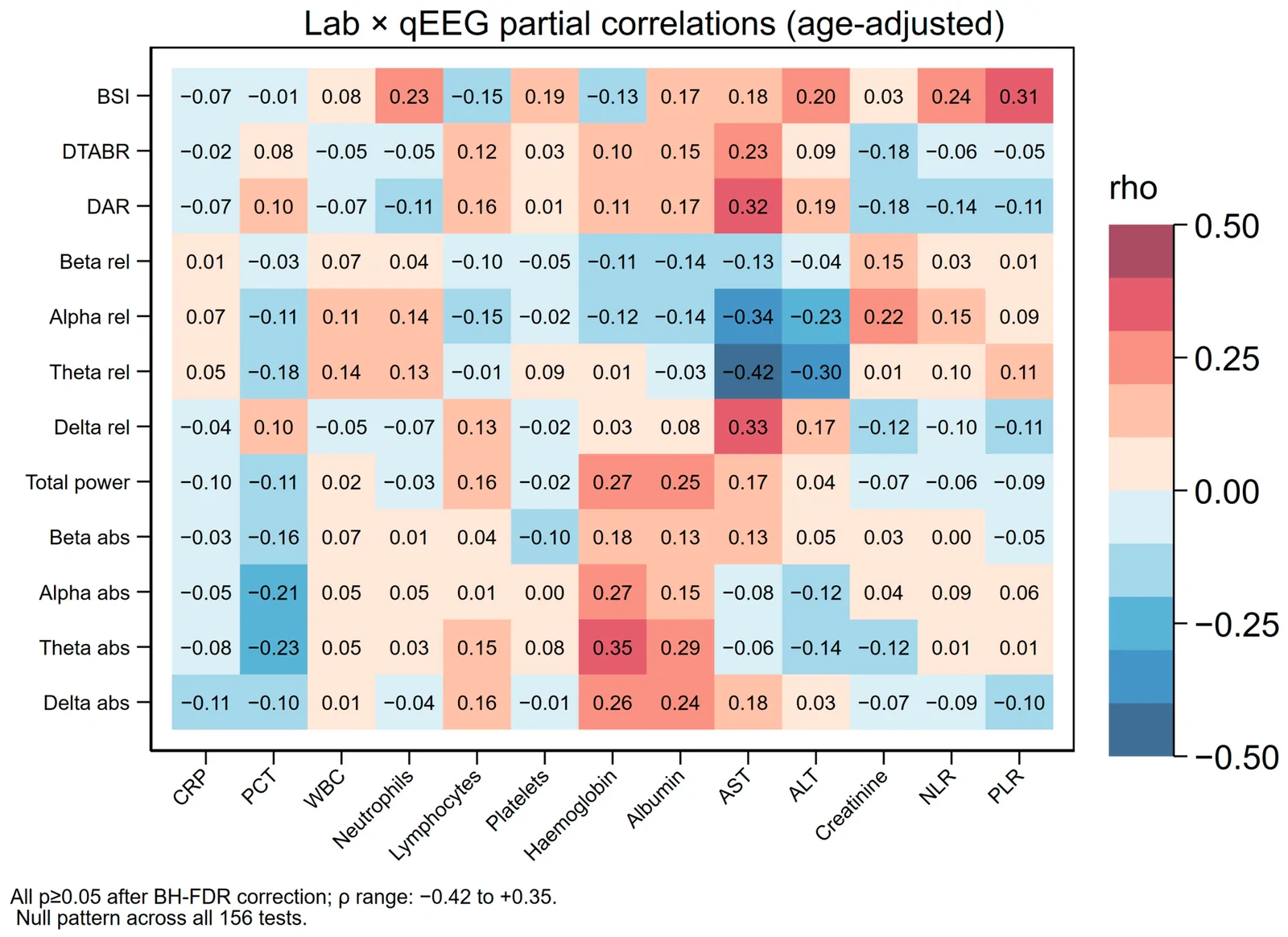
Grid A: age-adjusted partial Spearman correlation matrix between 13 laboratory markers (columns) and 12 quantitative spectral qEEG metrics (rows), spectral cohort (*n* = 71; effective *n* per cell 52–69). Cells show the age-adjusted partial Spearman ρ; the colour scale is symmetric from −0.5 to +0.5. Inflammatory-marker columns (CRP, PCT, leucocytes, neutrophils) were uniformly weak across all qEEG metrics. BH, Benjamini–Hochberg; CRP, C-reactive protein; PCT, procalcitonin; DAR, delta/alpha ratio; DTABR, (delta + theta)/(alpha + beta) ratio; BSI, brain symmetry index; NLR, neutrophil-to-lymphocyte ratio; PLR, platelet-to-lymphocyte ratio.

**Figure 5:**
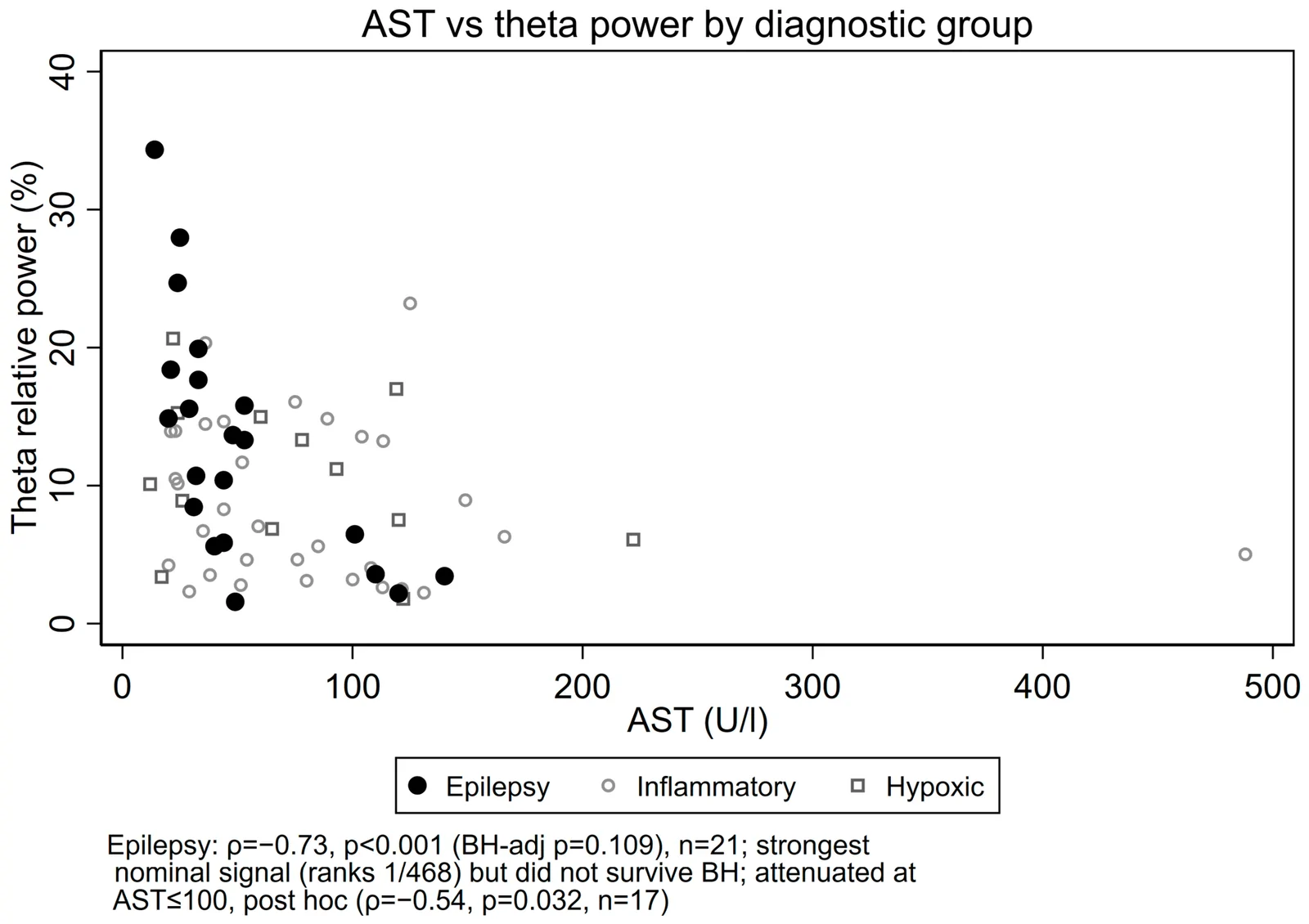
Association between AST and relative theta power stratified by diagnostic group (epilepsy, *n* = 23; inflammatory, *n* = 33; hypoxic–ischaemic, *n* = 13; spectral cohort). Age-adjusted partial Spearman correlation in the epilepsy subgroup: ρ = −0.73, raw *p*-value < 0.001, BH-adjusted *p*-value = 0.109, *n* = 21; AST ≤ 100 U/L sensitivity: ρ = −0.54, *p*-value = 0.032, *n* = 17. Points show unadjusted values; the reported statistic is the age-adjusted partial Spearman correlation. This association did not survive global BH-FDR correction and is reported as hypothesis-generating only. Between-group qEEG comparisons showed no result surviving BH correction (smallest BH-adjusted *p*-value = 0.13 for absolute alpha power); alpha power medians: epilepsy 4.55, inflammatory 1.97, hypoxic 2.31 µV^2^ (nominal Kruskal–Wallis *p*-value = 0.011, BH-adjusted *p*-value = 0.127). AST, aspartate aminotransferase.

**Table 1:** Characteristics of the spectral and cortical silence cohorts.

Characteristic	Spectral Cohort (Synek 1–4)	Cortical Silence (Synek 5)	*p*-Value
Age, months	24.0 (7.0–99.0)	82.5 (25.0–134.0)	0.010
Weight, kg	10.0 (3.7–16.0)	26.0 (14.0–48.5)	0.001
Male sex, *n* (%)	47 (66.2)	18 (69.2)	1.000
In-hospital death, *n* (%)	5 (7.0)	11 (42.3)	<0.001
Length of stay, days	25.0 (16.0–42.0)	9.0 (7.0–16.0)	<0.001
CRP, mg/dL	0.55 (0.28–2.65)	12.34 (4.39–18.23)	<0.001
PCT, ng/mL	0.24 (0.13–1.50)	3.22 (0.76–15.09)	<0.001
Leukocytes, ×10^3^/µL	9.64 (7.18–13.49)	9.70 (7.55–12.99)	0.955
Platelets, ×10^3^/µL	306 (179–444)	121 (69–237)	0.001
Albumin, g/dL	3.50 (3.20–3.70)	2.95 (2.45–3.60)	0.024
AST, U/L	51.4 (29.0–104.0)	111.0 (55.0–210.0)	0.001
Creatinine, mg/dL	0.27 (0.20–0.50)	0.60 (0.39–1.22)	0.001

Values are median (IQR) unless stated. *p*-value from Mann–Whitney U for continuous variables and Fisher exact for proportions. Missingness reflected routine clinical sampling: CRP *n* = 69/24 and PCT *n* = 58/24 in the spectral/cortical silence cohorts, respectively; weight *n* = 37/20; albumin *n* = 52/12. CRP, C-reactive protein; PCT, procalcitonin; AST, aspartate aminotransferase.

**Table 2:** Inflammatory and systemic severity markers by Synek EEG grade.

Marker	Grade 1	Grade 2	Grade 3	Grade 4	Grade 5
CRP, mg/dL	1.22 (0.42–3.21)	0.38 (0.20–1.15)	0.60 (0.23–2.37)	0.64 (0.41–3.81)	12.34 (4.39–18.23)
PCT, ng/mL	0.24 (0.14–0.78)	0.18 (0.10–0.50)	0.25 (0.15–2.10)	0.39 (0.13–3.43)	3.22 (0.76–15.09)
AST, U/L	36 (26–75)	46 (24–60)	101 (44–120)	38 (31–78)	111 (55–210)
Platelets, ×10^3^/µL	254 (189–446)	347 (238–455)	296 (171–418)	270 (140–374)	121 (69–237)
*n*	6–9	15–18	21–24	16–19	23–24

Values are median (IQR). The *n* row gives the range of available observations across the four markers shown in this table per grade. Grade 5 corresponds to the cortical silence cohort. Note the discontinuity from grades 1–4 to grade 5 for CRP and PCT.

## Data Availability

The data underlying this study contain identifiable clinical information from a paediatric intensive care population and cannot be made publicly available, in accordance with the terms of the institutional Ethics Committee approval (4920/15 April 2026), which authorised a retrospective-records waiver of informed consent for the stated research purpose only. De-identified data may be made available by the corresponding author upon reasonable request, subject to institutional and Ethics Committee approval.

## Supplementary Materials

The following supporting information can be downloaded at: https://www.mdpi.com/article/10.3390/brainsci16101012/s1, Supplementary Materials S1: STROBE Statement Checklist; Supplementary Materials S2: Detailed statistical specification.

## Abbreviations

The following abbreviations are used in this manuscript:

ALT	alanine aminotransferase
AST	aspartate aminotransferase
BH	Benjamini–Hochberg
BSI	brain symmetry index
CI	confidence interval
CRP	C-reactive protein
CSF	cerebrospinal fluid
DAR	delta/alpha ratio
DTABR	(delta + theta)/(alpha + beta) ratio
EDF	European Data Format
EEG	electroencephalography
FDR	false discovery rate
GFAP	glial fibrillary acidic protein
ICA	independent component analysis
IQR	interquartile range
NfL	neurofilament light chain
NLR	neutrophil-to-lymphocyte ratio
OR	odds ratio
PCT	procalcitonin
PICU	paediatric intensive care unit
PLR	platelet-to-lymphocyte ratio
qEEG	quantitative electroencephalography
S100B	S100 calcium-binding protein B
SAE	sepsis-associated encephalopathy
STROBE	Strengthening the Reporting of Observational Studies in Epidemiology
WBC	white blood cell
