# Supplementary material for: Procalcitonin and C-Reactive Protein Are Elevated at the Threshold of Cortical Electrocerebral Silence but Do Not Track Graded EEG Severity in Critically Ill Children: A Single-Centre Retrospective Cohort Study
Source: Brain Sci. 2026 Sep 24;16(10):1012. doi: 10.3390/brainsci16101012 (PMC13702215; doi:10.3390/brainsci16101012)
Supplement: Supplementary file 1 [file brainsci-16-01012-s001.zip › Supplementary_material_S1.pdf]

STROBE Statement Checklist

Item No.	Section/Topic	STROBE recommendation	Manuscript location
1(a)	Title and abstract	Indicate the study's design with a commonly used term in the title or the abstract.	Title; Abstract (Methods)
1(b)	Title and abstract	Provide in the abstract an informative and balanced summary of what was done and what was found.	Abstract (Background, Methods, Results, Conclusions)
2	Introduction	Explain the scientific background and rationale for the investigation being reported.	Section 1 Introduction, paragraphs 1–3
3	Introduction	State specific objectives, including any prespecified hypotheses.	Section 1 Introduction, final paragraph
4	Methods	Present key elements of study design early in the paper.	Methods — 2.1 Study design and setting
5	Methods	Describe the setting, locations, and relevant dates, including periods of recruitment, exposure, follow-up, and data collection.	Methods — 2.1 Study design and setting; 2.2 Participants; 2.5 Inflammatory biomarkers and laboratory variables; 2.6 EEG acquisition and sedation
6(a)	Methods	Give the eligibility criteria, and the sources and methods of selection of participants. Describe methods of follow-up.	Methods — 2.2 Participants; 2.3 Cohort stratification. Results — 3.1 Study population. Figure 1
6(b)	Methods	For matched studies, give matching criteria and number of exposed and unexposed.	Not applicable
7	Methods	Clearly define all outcomes, exposures, predictors, potential confounders, and effect modifiers. Give diagnostic criteria, if applicable.	Methods — 2.4 Clinical data; 2.5 Inflammatory biomarkers and laboratory variables; 2.6 EEG acquisition and sedation; 2.7 EEG visual review and Synek grading; 2.8 Quantitative EEG (qEEG) processing; 2.9 Statistical analysis
8	Methods	For each variable of interest, give sources of data and details of methods of assessment or measurement. Describe comparability of assessment methods if there is more than one group.	Methods — 2.4 Clinical data; 2.5 Inflammatory biomarkers and laboratory variables; 2.6 EEG acquisition and sedation; 2.7 EEG visual review and Synek grading; 2.8 Quantitative EEG (qEEG) processing
9	Methods	Describe any efforts to address potential sources of bias.	Methods — 2.6 EEG acquisition and sedation; 2.7 EEG visual review and Synek grading; 2.8 Quantitative EEG (qEEG) processing; 2.9 Statistical analysis. Results — 3.7 Robustness, sensitivity and inter-rater reliability. Section 4, Limitations
10	Methods	Explain how the study size was arrived at.	Methods — 2.2 Participants; 2.3 Cohort stratification; 2.9 Statistical analysis. Results — 3.1 Study population
11	Methods	Explain how quantitative variables were handled in the analyses. If applicable, describe which groupings were chosen and why.	Methods — 2.3 Cohort stratification; 2.8 Quantitative EEG (qEEG) processing; 2.9 Statistical analysis
12(a)	Methods	Describe all statistical methods, including those used to control for confounding.	Methods — 2.9 Statistical analysis. Supplementary Materials S2 (Sections S2.1–S2.3)
12(b)	Methods	Describe any methods used to examine subgroups and interactions.	Methods — 2.9 Statistical analysis. Supplementary Materials S2 (Section S2.5)
12(c)	Methods	Explain how missing data were addressed.	Methods — 2.5 Inflammatory biomarkers and laboratory variables; 2.9 Statistical analysis. Results — Table 1 footnote; 3.3 Inflammatory markers and the threshold of

Item No.	Section/Topic	STROBE recommendation	Manuscript location
			cortical electrocerebral silence; 3.4 Inflammatory markers and quantitative spectral qEEG
12(d)	Methods	If applicable, explain how loss to follow-up was addressed.	Not applicable
12(e)	Methods	Describe any sensitivity analyses.	Methods — 2.9 Statistical analysis. Results — 3.6 Exploratory analyses; 3.7 Robustness, sensitivity and inter-rater reliability. Supplementary Materials S2 (Section S2.7)
13(a)	Results	Report numbers of individuals at each stage of study, e.g., numbers potentially eligible, examined for eligibility, confirmed eligible, included in the study, completing follow-up, and analysed.	Results — 3.1 Study population. Figure 1. Methods — 2.3 Cohort stratification
13(b)	Results	Give reasons for non-participation at each stage.	Methods — 2.2 Participants; 2.3 Cohort stratification. Figure 1
13(c)	Results	Consider use of a flow diagram.	Figure 1
14(a)	Results	Give characteristics of study participants and information on exposures and potential confounders.	Results — 3.1 Study population; 3.2 Cohort characteristics. Tables 1 and 2
14(b)	Results	Indicate number of participants with missing data for each variable of interest.	Table 1 footnote; Table 2 (n row); Results — 3.3 Inflammatory markers and the threshold of cortical electrocerebral silence; 3.4 Inflammatory markers and quantitative spectral qEEG
14(c)	Results	Summarise follow-up time, e.g., average and total amount.	Not applicable — single-timepoint retrospective design without longitudinal follow-up. Length of PICU stay is reported in Results — 3.2 Cohort characteristics and Table 1
15	Results	Report numbers of outcome events or summary measures over time.	Results — 3.1 Study population; 3.8 Outcome. Table 1 (in-hospital death); Table 2
16(a)	Results	Give unadjusted estimates and, if applicable, confounder-adjusted estimates and their precision. Make clear which confounders were adjusted for and why they were included.	Results — 3.3 Inflammatory markers and the threshold of cortical electrocerebral silence; 3.7 Robustness, sensitivity and inter-rater reliability; 3.8 Outcome. Methods — 2.9 Statistical analysis. Supplementary Materials S2 (Section S2.6)
16(b)	Results	Report category boundaries when continuous variables were categorized.	Methods — 2.3 Cohort stratification; 2.5 Inflammatory biomarkers and laboratory variables; 2.7 EEG visual review and Synek grading; 2.8 Quantitative EEG (qEEG) processing; 2.9 Statistical analysis. Results — Tables 1 and 2
16(c)	Results	If relevant, consider translating estimates of relative risk into absolute risk for a meaningful time period.	Not applicable
17	Results	Report other analyses done, e.g., analyses of subgroups and interactions, and sensitivity analyses.	Results — 3.4 Inflammatory markers and quantitative spectral qEEG (montage-homogeneous 10–20 subset); 3.5 Post hoc analyses; 3.6 Exploratory analyses; 3.7 Robustness, sensitivity and inter-rater reliability; 3.8 Outcome. Figures 4 and 5. Supplementary Materials S2
18	Discussion	Summarise key results with reference to study objectives.	Section 4 Discussion, first two paragraphs; Section 5 Conclusions

Item No.	Section/Topic	STROBE recommendation	Manuscript location
19	Discussion	Discuss limitations of the study, taking into account sources of potential bias or imprecision. Discuss both direction and magnitude of any potential bias.	Section 4, Limitations (all four paragraphs); Discussion, final paragraph
20	Discussion	Give a cautious overall interpretation of results considering objectives, limitations, multiplicity of analyses, results from similar studies, and other relevant evidence.	Section 4 Discussion; Section 5 Conclusions
21	Discussion	Discuss the generalisability/external validity of the study results.	Section 4, Discussion, final paragraph; Limitations, first and third paragraphs
22	Other information	Give the source of funding and the role of the funders for the present study and, if applicable, for the original study on which the present article is based.	Funding; Acknowledgments
