# Supplementary material for: Procalcitonin and C-Reactive Protein Are Elevated at the Threshold of Cortical Electrocerebral Silence but Do Not Track Graded EEG Severity in Critically Ill Children: A Single-Centre Retrospective Cohort Study
Source: Brain Sci. 2026 Sep 24;16(10):1012. doi: 10.3390/brainsci16101012 (PMC13702215; doi:10.3390/brainsci16101012)
Supplement: Supplementary file 1 [file brainsci-16-01012-s001.zip › Supplementary_material_S2.pdf]

# Supplementary material S2. Detailed statistical specification

*Procalcitonin and C-reactive protein are elevated at the threshold of cortical electrocerebral silence but do not track graded EEG severity in critically ill children: a single-centre retrospective cohort study*

All analyses were performed in Stata 19.5 BE. This document specifies analyses summarised in the main text and reports in full the laboratory × qEEG grid (Grid A) and its sensitivity analyses.

## S2.1 Analysis families and multiplicity control

The analysis plan was defined after data collection and was not prospectively registered. The primary and secondary families were specified before their results were inspected; all other analyses were supporting, exploratory or post hoc, as indicated below. Benjamini–Hochberg (BH) correction was applied within each family and not across families.

Primary: Spearman correlations of PCT and CRP with Synek grade across the combined cohort (grades 1–5). BH across 2.

Secondary: five age-adjusted partial Spearman correlations between inflammatory markers and spectral qEEG in the spectral cohort. BH across 5.

Supporting analyses of the primary association: Mann–Whitney comparison of cortical silence with preserved activity; Spearman correlations within grades 1–4; logistic regression (Section S2.6). Not corrected for multiplicity.

Post hoc: AST and platelet count against Synek grade, selected after inspection of the cohort differences. BH across 2.

Exploratory Grid A: 13 laboratory markers × 12 qEEG metrics, 156 age-adjusted partial correlations. BH across 156.

Exploratory Grid B: qEEG metrics against Synek grade (Spearman, Kruskal–Wallis and quadratic-term tests). BH within each of the three sub-families.

Post hoc diagnostic subgroups: 468 age-adjusted partial correlations (3 groups × 13 markers × 12 metrics). BH across 468. The AST ≤100 U/l restriction was defined after inspection of the scatterplot.

Sensitivity analyses: relative beta ≤25%, 3×IQR outlier trimming, full 10–20 subset and exclusion of neonates (Section S2.7).

Tests were two-tailed at $\alpha=0.05$. BH correction was implemented directly in Stata.

## S2.2 Composite inflammatory score

The composite inflammatory score was derived in three steps.

1. Log-transformation: natural log of each marker after adding a constant of 0.01, to accommodate PCT values at or near zero: ln(PCT + 0.01) and ln(CRP + 0.01).
2. Robust standardisation: each log-transformed marker was centred on its median and scaled by its interquartile range, $z = (\ln x - \text{median}) / (p75 - p25)$. The median and IQR were computed on the combined cohort (spectral plus cortical-silence subsets, n=97), not on the spectral subset alone, so that the two subsets are placed on a common scale. The denominator is the raw interquartile range and is not rescaled to a normal-distribution equivalent.

3. Combination: the unweighted mean of the two robust z-scores. Equal weights were used because there is no external criterion for weighting CRP relative to PCT, and weights estimated against the EEG outcomes in the same dataset would have been circular. Both markers were on a common robust scale, and each was also analysed individually in the secondary analyses.

Two versions were computed, differing only in how records with a single available marker were treated:

- Primary composite: defined only when both PCT and CRP were available; records with one marker were set to missing. Effective n = 57 in the spectral cohort. This is the version reported in the main analysis.
- Permissive composite: the mean of whichever z-scores were available, so that a record with a single marker takes the value of that marker's z-score; missing only when both markers were absent. Effective n = 70 in the spectral cohort. Reported alongside the primary composite as a sensitivity estimate ($\rho=-0.11$, p-value $=0.37$).

## S2.3 Partial correlation method

All age-adjusted associations were estimated in Stata as partial Spearman correlations by rank residualisation. For each pair, the analysis was restricted to observations with complete data on both variables and age, and all three variables were ranked within that sample (egen rank, average ranks for ties). The ranks of each variable were regressed on the rank of age (regress), the residuals were saved (predict, resid), and the Pearson correlation between the two sets of residuals (correlate) was taken as the partial Spearman coefficient. The p-value was obtained from a t distribution with $n - 2 - k$ degrees of freedom, where k is the number of covariates (k=1 for age). Confidence intervals were obtained by the Fisher z-transform as 95% CI = $\tanh(\operatorname{arctanh}(\rho) \pm 1.96/\sqrt{(n - 3 - k)})$, that is with standard error $1/\sqrt{(n - 4)}$ for the age-adjusted partial correlations (k=1) and $1/\sqrt{(n - 3)}$ for unadjusted Spearman correlations (k=0). Because the number of complete observations differed between pairs, the effective n is reported for each coefficient.

Spearman rather than Pearson correlations were used throughout because several laboratory markers and spectral power values were strongly right-skewed and contained extreme values that are clinically real rather than erroneous.

## S2.4 Grid A and Grid B specification

Grid A crossed 13 laboratory markers with 12 quantitative spectral qEEG metrics in the spectral cohort (n=71), giving 156 age-adjusted partial Spearman correlations.

Laboratory markers (13): CRP, PCT, leucocytes, absolute neutrophil count, absolute lymphocyte count, neutrophil-to-lymphocyte ratio (NLR), platelet-to-lymphocyte ratio (PLR), platelets, haemoglobin, albumin, AST, ALT, creatinine.

qEEG metrics (12): absolute power in delta, theta, alpha and beta; total spectral power; relative power in delta, theta, alpha and beta; delta–alpha ratio (DAR); delta–theta–alpha–beta ratio (DTABR); brain symmetry index (BSI).

Peak alpha frequency was excluded from Grid A because it was non-identifiable in 60 of 71 recordings (84.5%), the spectral maximum falling at the lower band boundary.
Effective n per cell ranged from 52 to 69, reflecting routine clinical sampling of the laboratory markers rather than any analytic exclusion.
Grid B tested each of the same 12 qEEG metrics against Synek grade within the spectral cohort (grades 1–4) using three approaches: monotonic Spearman correlation, Kruskal–Wallis comparison across grades, and a quadratic term to detect non-monotonic relationships. The quadratic term was included because the spectral slowing indices were expected not to increase monotonically with visual severity: total power collapses at the most suppressed preserved grade, so DTABR and DAR peak at grade 3 and fall at grade 4.

## S2.5 Diagnostic-subgroup correlations

Grid A was repeated within each of the three diagnostic categories (epilepsy (n=23), inflammatory brain injury (n=33) and hypoxic–ischaemic injury (n=13)) giving 468 age-adjusted partial correlations, BH-corrected across all 468. Two patients with trauma were excluded from subgroup analyses on sample-size grounds. Twenty pairs were nominally significant at raw p-value <0.05 and none survived BH correction. The strongest was AST versus relative theta power in the epilepsy subgroup (ρ=−0.73, raw p-value <0.001, BH-adjusted p-value =0.109, n=21), with a post hoc sensitivity estimate restricted to AST ≤100 U/l, defined after inspection of the scatterplot. Descriptive inflammatory-marker profiles by aetiology in the combined cohort are given in Table S2.4.

## S2.6 Robustness models

The contrast between Synek 5 and Synek 1–4 was additionally modelled by logistic regression on ln-transformed markers under three adjustment sets. Each model included 24 children with cortical silence and an available marker value:

| Model | Adjustment set | Parameters | Events per parameter | Note |
|---|---|---|---|---|
| M0 | None | 1 | 24 | Unadjusted |
| M1 | Age + creatinine | 3 | 8 | Adjusted estimate reported in the main text |
| M2 | Age + creatinine + diagnostic category | 6 | 4 | Exploratory; not interpreted |

In M2, the marker estimates were PCT OR 1.95 (95% CI 1.28–2.95) and CRP OR 2.25 (1.43–3.53). The diagnostic-category coefficients were unstable, with near-complete separation for trauma (6 of 7 children with cortical silence in the PCT model; OR 14.20, 95% CI 0.82–244.6), and are not interpreted. No model adjusted for a

validated global illness-severity score, as none was reliably available in the retrospective record. This remains the principal source of residual confounding.

## S2.7 Sensitivity analyses

Four sensitivity analyses were performed. Narrower biomarker–EEG intervals could not be tested because exact sampling times were not recorded.

- Pharmacological beta exclusion: recordings with relative beta power above 25% were excluded as a proxy for benzodiazepine-induced beta activity, and Grid A was repeated (n=2 recordings excluded).
- Outlier trimming:for each qEEG metric, values outside median ± 3 × IQR within the spectral cohort were flagged. Trimming was applied per metric and per pair: an observation was excluded only from those pairs involving the metric on which it was an outlier, not from the analysis as a whole. Effective n therefore varies by cell.
- Montage-homogeneous subset: analyses restricted to children aged ≥8 months, recorded with the standard international 10–20 montage (n=52). The five secondary correlations were re-estimated in this subset: CRP × absolute delta ρ=−0.160 (95% CI −0.423 to 0.126; n=50; BH-adjusted p-value =0.807); CRP × DTABR ρ=−0.136 (−0.402 to 0.151; n=50; BH-adjusted p-value =0.807); composite inflammatory score × total power ρ=−0.114 (−0.411 to 0.205; n=41; BH-adjusted p-value =0.807); PCT × DTABR ρ=+0.068 (−0.245 to 0.368; n=42; BH-adjusted p-value =0.842); PCT × absolute delta ρ=−0.032 (−0.336 to 0.278; n=42; BH-adjusted p-value =0.842). The permissive composite gave ρ=−0.094 (n=51, p-value =0.518). The full laboratory × qEEG grid repeated in this subset is given in Table S2.3. Because montage was assigned by age, a direct comparison of spectral values between montages cannot separate montage-related variance from maturational differences; montage was therefore addressed through age adjustment and this restricted analysis.
- Exclusion of neonates : infants aged <1 month (n=4 in the combined cohort) were excluded to address the possibility that age-related normalisation of spectral metrics drove the associations. The primary association was unchanged (PCT × Synek ρ=+0.500, p-value =$2.7\times10^{-6}$, n=79; age- and creatinine-adjusted ρ=+0.468, p-value =$2.3\times10^{-5}$, n=77; CRP × Synek ρ=+0.406, p-value =$7.7\times10^{-5}$, n=89; adjusted ρ=+0.333, p-value =0.002, n=86), as was the secondary null (all five correlations |ρ|≤0.137, all p-value ≥0.330; effective n 54–65). This analysis does not correct for age effects across the full paediatric range, which remains a limitation.

Full results for the first two are given in Table S2.2. In neither did any pair survive BH correction (smallest BH-adjusted p-value = 0.103 and 0.077 respectively).

## S2.8 Power

No a priori sample size calculation was performed, as the sample was fixed by the retrospective design. After the analyses, the smallest correlation detectable with 80% power (two-sided α=0.05) was calculated for each available sample size on the

Fisher z scale: $\rho_{80} = \tanh((z_{0.80} + z_{0.975})/\sqrt{(n - 3 - k)})$, with k=1 for the age-adjusted partial correlations and k=0 for unadjusted Spearman correlations.

| Analysis | n | Correlation detectable with 80% power |
|---|---|---|
| Primary: PCT × Synek grade | 82 | 0.31 |
| Primary: CRP × Synek grade | 93 | 0.29 |
| Secondary and within grades 1–4: PCT | 58 | 0.36 |
| Secondary and within grades 1–4: CRP | 69 | 0.33 |
| Secondary: composite score × total power | 57 | 0.37 |
| Secondary, full 10–20 subset | 41–50 | 0.39–0.43 |
| Epilepsy subgroup: AST × relative theta | 21 | 0.59 |

Power was evaluated at the rounded coefficients passed to the power routine (PCT × DTABR, 0.085 versus 0.084 as reported; CRP × absolute delta, 0.110, entered as an absolute value; AST × relative theta, 0.742 versus 0.733 as reported in S2.5 and Figure 5). These differences do not materially affect the power estimates, and the correlation detectable with 80% power depends only on n and is unaffected.

The secondary analyses were therefore powered only for moderate-to-strong effects: at the effective n of 57–69 available in the spectral cohort, 80% power corresponded to $\rho \approx 0.33$–0.37, and in the montage-homogeneous subset to $\rho \approx 0.39$–0.43. Weak true associations below these thresholds cannot be excluded.

## Table S2.1. Grid A: laboratory markers × quantitative spectral qEEG metrics, spectral cohort

Age-adjusted partial Spearman correlations, n=71 (effective n per cell 52–69). Confidence intervals by Fisher z-transform with standard error $1/\sqrt{(n-4)}$. Benjamini–Hochberg correction applied across all 156 tests. No pair survived correction; the smallest BH-adjusted p-value was 0.077. Coefficients ranged from −0.417 to +0.353.

| Marker | qEEG metric | ρ | 95% CI | n | p-value (raw) | p-value (BH) |
|---|---|---|---|---|---|---|
| CRP | Delta, absolute | -0.110 | -0.339 to 0.132 | 69 | 0.373 | 0.867 |
| CRP | Theta, absolute | -0.082 | -0.314 to 0.160 | 69 | 0.508 | 0.881 |
| CRP | Alpha, absolute | -0.046 | -0.281 to 0.195 | 69 | 0.711 | 0.941 |
| CRP | Beta, absolute | -0.031 | -0.267 to 0.209 | 69 | 0.804 | 0.956 |
| CRP | Total power | -0.099 | -0.330 to 0.143 | 69 | 0.422 | 0.867 |
| CRP | Delta, relative | -0.041 | -0.277 to 0.199 | 69 | 0.739 | 0.941 |
| CRP | Theta, relative | 0.052 | -0.189 to 0.287 | 69 | 0.674 | 0.941 |
| CRP | Alpha, relative | 0.075 | -0.167 to 0.308 | 69 | 0.544 | 0.913 |
| CRP | Beta, relative | 0.012 | -0.227 to 0.250 | 69 | 0.921 | 0.963 |
| CRP | DAR | -0.067 | -0.301 to 0.174 | 69 | 0.586 | 0.916 |
| CRP | DTABR | -0.023 | -0.260 to 0.216 | 69 | 0.851 | 0.963 |
| CRP | BSI | -0.068 | -0.301 to 0.174 | 69 | 0.583 | 0.916 |
| PCT | Delta, absolute | -0.104 | -0.355 to 0.161 | 58 | 0.440 | 0.869 |
| PCT | Theta, absolute | -0.229 | -0.462 to 0.034 | 58 | 0.087 | 0.714 |
| PCT | Alpha, absolute | -0.214 | -0.450 to 0.049 | 58 | 0.110 | 0.815 |
| PCT | Beta, absolute | -0.164 | -0.407 to 0.101 | 58 | 0.224 | 0.867 |
| PCT | Total power | -0.107 | -0.358 to 0.158 | 58 | 0.426 | 0.867 |
| PCT | Delta, relative | 0.102 | -0.163 to 0.353 | 58 | 0.451 | 0.872 |
| PCT | Theta, relative | -0.181 | -0.421 to 0.084 | 58 | 0.179 | 0.867 |
| PCT | Alpha, relative | -0.112 | -0.362 to 0.153 | 58 | 0.405 | 0.867 |
| PCT | Beta, relative | -0.033 | -0.292 to 0.229 | 58 | 0.805 | 0.956 |
| PCT | DAR | 0.101 | -0.164 to 0.352 | 58 | 0.454 | 0.872 |
| PCT | DTABR | 0.084 | -0.181 to 0.337 | 58 | 0.535 | 0.907 |
| PCT | BSI | -0.011 | -0.271 to 0.250 | 58 | 0.933 | 0.963 |
| Leucocytes | Delta, absolute | 0.011 | -0.232 to 0.252 | 67 | 0.931 | 0.963 |
| Leucocytes | Theta, absolute | 0.053 | -0.192 to 0.291 | 67 | 0.674 | 0.941 |
| Leucocytes | Alpha, absolute | 0.049 | -0.195 to 0.288 | 67 | 0.696 | 0.941 |
| Leucocytes | Beta, absolute | 0.072 | -0.174 to 0.308 | 67 | 0.568 | 0.916 |
| Leucocytes | Total power | 0.020 | -0.224 to 0.260 | 67 | 0.876 | 0.963 |
| Leucocytes | Delta, relative | -0.050 | -0.289 to 0.194 | 67 | 0.688 | 0.941 |
| Leucocytes | Theta, relative | 0.140 | -0.105 to 0.370 | 67 | 0.261 | 0.867 |
| Leucocytes | Alpha, relative | 0.106 | -0.140 to 0.339 | 67 | 0.398 | 0.867 |
| Leucocytes | Beta, relative | 0.071 | -0.174 to 0.308 | 67 | 0.570 | 0.916 |
| Leucocytes | DAR | -0.068 | -0.305 to 0.177 | 67 | 0.588 | 0.916 |
| Leucocytes | DTABR | -0.052 | -0.291 to 0.192 | 67 | 0.676 | 0.941 |
| Leucocytes | BSI | 0.084 | -0.161 to 0.320 | 67 | 0.502 | 0.880 |
| Neutrophils | Delta, absolute | -0.043 | -0.282 to 0.201 | 67 | 0.734 | 0.941 |
| Neutrophils | Theta, absolute | 0.028 | -0.215 to 0.268 | 67 | 0.823 | 0.956 |
| Neutrophils | Alpha, absolute | 0.054 | -0.191 to 0.292 | 67 | 0.670 | 0.941 |
| Neutrophils | Beta, absolute | 0.010 | -0.233 to 0.251 | 67 | 0.937 | 0.963 |
| Neutrophils | Total power | -0.028 | -0.268 to 0.215 | 67 | 0.823 | 0.956 |
| Neutrophils | Delta, relative | -0.067 | -0.304 to 0.178 | 67 | 0.593 | 0.916 |
| Neutrophils | Theta, relative | 0.132 | -0.114 to 0.363 | 67 | 0.290 | 0.867 |
| Neutrophils | Alpha, relative | 0.136 | -0.110 to 0.366 | 67 | 0.276 | 0.867 |
| Neutrophils | Beta, relative | 0.039 | -0.205 to 0.278 | 67 | 0.757 | 0.953 |
| Neutrophils | DAR | -0.106 | -0.340 to 0.139 | 67 | 0.395 | 0.867 |
| Neutrophils | DTABR | -0.047 | -0.286 to 0.197 | 67 | 0.709 | 0.941 |
| Neutrophils | BSI | 0.226 | -0.017 to 0.444 | 67 | 0.068 | 0.666 |
| Lymphocytes | Delta, absolute | 0.165 | -0.082 to 0.393 | 66 | 0.189 | 0.867 |
| Lymphocytes | Theta, absolute | 0.154 | -0.094 to 0.383 | 66 | 0.222 | 0.867 |
| Lymphocytes | Alpha, absolute | 0.014 | -0.231 to 0.257 | 66 | 0.913 | 0.963 |
| Lymphocytes | Beta, absolute | 0.042 | -0.204 to 0.283 | 66 | 0.742 | 0.941 |
| Lymphocytes | Total power | 0.159 | -0.088 to 0.388 | 66 | 0.205 | 0.867 |
| Lymphocytes | Delta, relative | 0.130 | -0.117 to 0.363 | 66 | 0.301 | 0.867 |
| Lymphocytes | Theta, relative | -0.007 | -0.251 to 0.237 | 66 | 0.956 | 0.974 |
| Lymphocytes | Alpha, relative | -0.150 | -0.380 to 0.097 | 66 | 0.232 | 0.867 |

| | | | | | | |
|---|---|---|---|---|---|---|
| Lymphocytes | Beta, relative | -0.100 | -0.335 to 0.148 | 66 | 0.430 | 0.867 |
| Lymphocytes | DAR | 0.159 | -0.089 to 0.388 | 66 | 0.207 | 0.867 |
| Lymphocytes | DTABR | 0.121 | -0.127 to 0.354 | 66 | 0.338 | 0.867 |
| Lymphocytes | BSI | -0.155 | -0.384 to 0.093 | 66 | 0.219 | 0.867 |
| NLR | Delta, absolute | -0.086 | -0.323 to 0.161 | 66 | 0.497 | 0.880 |
| NLR | Theta, absolute | 0.006 | -0.238 to 0.249 | 66 | 0.963 | 0.975 |
| NLR | Alpha, absolute | 0.090 | -0.158 to 0.326 | 66 | 0.478 | 0.880 |
| NLR | Beta, absolute | 0.000 | -0.244 to 0.244 | 66 | 0.998 | 0.998 |
| NLR | Total power | -0.065 | -0.304 to 0.182 | 66 | 0.607 | 0.920 |
| NLR | Delta, relative | -0.099 | -0.335 to 0.149 | 66 | 0.433 | 0.867 |
| NLR | Theta, relative | 0.100 | -0.148 to 0.335 | 66 | 0.430 | 0.867 |
| NLR | Alpha, relative | 0.146 | -0.102 to 0.376 | 66 | 0.246 | 0.867 |
| NLR | Beta, relative | 0.028 | -0.218 to 0.270 | 66 | 0.827 | 0.956 |
| NLR | DAR | -0.142 | -0.373 to 0.105 | 66 | 0.258 | 0.867 |
| NLR | DTABR | -0.057 | -0.297 to 0.190 | 66 | 0.653 | 0.941 |
| NLR | BSI | 0.236 | -0.009 to 0.454 | 66 | 0.059 | 0.666 |
| PLR | Delta, absolute | -0.104 | -0.339 to 0.144 | 66 | 0.412 | 0.867 |
| PLR | Theta, absolute | 0.010 | -0.235 to 0.253 | 66 | 0.938 | 0.963 |
| PLR | Alpha, absolute | 0.062 | -0.185 to 0.301 | 66 | 0.625 | 0.937 |
| PLR | Beta, absolute | -0.051 | -0.291 to 0.196 | 66 | 0.689 | 0.941 |
| PLR | Total power | -0.093 | -0.329 to 0.155 | 66 | 0.462 | 0.872 |
| PLR | Delta, relative | -0.113 | -0.347 to 0.135 | 66 | 0.371 | 0.867 |
| PLR | Theta, relative | 0.111 | -0.137 to 0.346 | 66 | 0.379 | 0.867 |
| PLR | Alpha, relative | 0.092 | -0.155 to 0.329 | 66 | 0.464 | 0.872 |
| PLR | Beta, relative | 0.011 | -0.233 to 0.254 | 66 | 0.930 | 0.963 |
| PLR | DAR | -0.109 | -0.344 to 0.138 | 66 | 0.387 | 0.867 |
| PLR | DTABR | -0.049 | -0.290 to 0.197 | 66 | 0.696 | 0.941 |
| PLR | BSI | 0.311 | 0.072 to 0.515 | 66 | 0.012 | 0.304 |
| Platelets | Delta, absolute | -0.010 | -0.251 to 0.233 | 67 | 0.938 | 0.963 |
| Platelets | Theta, absolute | 0.080 | -0.165 to 0.316 | 67 | 0.522 | 0.894 |
| Platelets | Alpha, absolute | 0.004 | -0.238 to 0.246 | 67 | 0.974 | 0.980 |
| Platelets | Beta, absolute | -0.101 | -0.335 to 0.144 | 67 | 0.419 | 0.867 |
| Platelets | Total power | -0.020 | -0.261 to 0.223 | 67 | 0.874 | 0.963 |
| Platelets | Delta, relative | -0.020 | -0.260 to 0.224 | 67 | 0.876 | 0.963 |
| Platelets | Theta, relative | 0.086 | -0.159 to 0.322 | 67 | 0.490 | 0.880 |
| Platelets | Alpha, relative | -0.017 | -0.258 to 0.226 | 67 | 0.891 | 0.963 |
| Platelets | Beta, relative | -0.051 | -0.289 to 0.193 | 67 | 0.685 | 0.941 |
| Platelets | DAR | 0.015 | -0.228 to 0.256 | 67 | 0.908 | 0.963 |
| Platelets | DTABR | 0.029 | -0.214 to 0.269 | 67 | 0.816 | 0.956 |
| Platelets | BSI | 0.185 | -0.059 to 0.409 | 67 | 0.136 | 0.841 |
| Haemoglobin | Delta, absolute | 0.259 | 0.018 to 0.472 | 67 | 0.036 | 0.557 |
| Haemoglobin | Theta, absolute | 0.353 | 0.122 to 0.548 | 67 | 0.004 | 0.269 |
| Haemoglobin | Alpha, absolute | 0.274 | 0.034 to 0.484 | 67 | 0.026 | 0.509 |
| Haemoglobin | Beta, absolute | 0.184 | -0.061 to 0.408 | 67 | 0.140 | 0.841 |
| Haemoglobin | Total power | 0.266 | 0.026 to 0.477 | 67 | 0.031 | 0.534 |
| Haemoglobin | Delta, relative | 0.029 | -0.214 to 0.270 | 67 | 0.815 | 0.956 |
| Haemoglobin | Theta, relative | 0.012 | -0.231 to 0.253 | 67 | 0.925 | 0.963 |
| Haemoglobin | Alpha, relative | -0.123 | -0.355 to 0.122 | 67 | 0.324 | 0.867 |
| Haemoglobin | Beta, relative | -0.111 | -0.343 to 0.135 | 67 | 0.377 | 0.867 |
| Haemoglobin | DAR | 0.111 | -0.134 to 0.344 | 67 | 0.374 | 0.867 |
| Haemoglobin | DTABR | 0.105 | -0.141 to 0.338 | 67 | 0.402 | 0.867 |
| Haemoglobin | BSI | -0.132 | -0.362 to 0.114 | 67 | 0.291 | 0.867 |
| Albumin | Delta, absolute | 0.245 | -0.033 to 0.487 | 52 | 0.084 | 0.714 |
| Albumin | Theta, absolute | 0.289 | 0.014 to 0.523 | 52 | 0.040 | 0.567 |
| Albumin | Alpha, absolute | 0.152 | -0.129 to 0.411 | 52 | 0.285 | 0.867 |
| Albumin | Beta, absolute | 0.128 | -0.153 to 0.390 | 52 | 0.370 | 0.867 |
| Albumin | Total power | 0.254 | -0.023 to 0.495 | 52 | 0.072 | 0.666 |
| Albumin | Delta, relative | 0.080 | -0.200 to 0.348 | 52 | 0.577 | 0.916 |
| Albumin | Theta, relative | -0.030 | -0.303 to 0.248 | 52 | 0.835 | 0.957 |
| Albumin | Alpha, relative | -0.137 | -0.398 to 0.144 | 52 | 0.337 | 0.867 |
| Albumin | Beta, relative | -0.140 | -0.400 to 0.141 | 52 | 0.328 | 0.867 |
| Albumin | DAR | 0.166 | -0.115 to 0.422 | 52 | 0.245 | 0.867 |
| Albumin | DTABR | 0.153 | -0.128 to 0.411 | 52 | 0.284 | 0.867 |
| Albumin | BSI | 0.166 | -0.115 to 0.422 | 52 | 0.245 | 0.867 |
| AST | Delta, absolute | 0.183 | -0.062 to 0.407 | 67 | 0.142 | 0.841 |
| AST | Theta, absolute | -0.055 | -0.293 to 0.189 | 67 | 0.659 | 0.941 |
| AST | Alpha, absolute | -0.084 | -0.320 to 0.161 | 67 | 0.502 | 0.880 |
| AST | Beta, absolute | 0.134 | -0.112 to 0.364 | 67 | 0.284 | 0.867 |
| AST | Total power | 0.174 | -0.071 to 0.399 | 67 | 0.163 | 0.867 |

| | | | | | | |
|---|---|---|---|---|---|---|
| AST | Delta, relative | 0.325 | 0.090 to 0.526 | 67 | 0.008 | 0.269 |
| AST | Theta, relative | -0.417 | -0.599 to -0.195 | 67 | <0.001 | 0.077 |
| AST | Alpha, relative | -0.339 | -0.537 to -0.106 | 67 | 0.005 | 0.269 |
| AST | Beta, relative | -0.127 | -0.358 to 0.119 | 67 | 0.309 | 0.867 |
| AST | DAR | 0.321 | 0.085 to 0.522 | 67 | 0.009 | 0.269 |
| AST | DTABR | 0.228 | -0.015 to 0.445 | 67 | 0.066 | 0.666 |
| AST | BSI | 0.184 | -0.060 to 0.408 | 67 | 0.138 | 0.841 |
| ALT | Delta, absolute | 0.035 | -0.209 to 0.275 | 67 | 0.780 | 0.956 |
| ALT | Theta, absolute | -0.140 | -0.369 to 0.106 | 67 | 0.262 | 0.867 |
| ALT | Alpha, absolute | -0.122 | -0.353 to 0.124 | 67 | 0.331 | 0.867 |
| ALT | Beta, absolute | 0.048 | -0.196 to 0.287 | 67 | 0.701 | 0.941 |
| ALT | Total power | 0.037 | -0.207 to 0.277 | 67 | 0.768 | 0.956 |
| ALT | Delta, relative | 0.166 | -0.079 to 0.392 | 67 | 0.182 | 0.867 |
| ALT | Theta, relative | -0.302 | -0.507 to -0.065 | 67 | 0.014 | 0.304 |
| ALT | Alpha, relative | -0.226 | -0.443 to 0.017 | 67 | 0.069 | 0.666 |
| ALT | Beta, relative | -0.043 | -0.282 to 0.201 | 67 | 0.730 | 0.941 |
| ALT | DAR | 0.189 | -0.056 to 0.412 | 67 | 0.129 | 0.841 |
| ALT | DTABR | 0.087 | -0.159 to 0.322 | 67 | 0.489 | 0.880 |
| ALT | BSI | 0.200 | -0.044 to 0.422 | 67 | 0.107 | 0.815 |
| Creatinine | Delta, absolute | -0.066 | -0.303 to 0.179 | 67 | 0.601 | 0.919 |
| Creatinine | Theta, absolute | -0.118 | -0.350 to 0.127 | 67 | 0.344 | 0.867 |
| Creatinine | Alpha, absolute | 0.045 | -0.200 to 0.284 | 67 | 0.722 | 0.941 |
| Creatinine | Beta, absolute | 0.032 | -0.212 to 0.272 | 67 | 0.800 | 0.956 |
| Creatinine | Total power | -0.074 | -0.310 to 0.172 | 67 | 0.557 | 0.916 |
| Creatinine | Delta, relative | -0.119 | -0.351 to 0.127 | 67 | 0.343 | 0.867 |
| Creatinine | Theta, relative | 0.014 | -0.229 to 0.255 | 67 | 0.910 | 0.963 |
| Creatinine | Alpha, relative | 0.222 | -0.021 to 0.441 | 67 | 0.073 | 0.666 |
| Creatinine | Beta, relative | 0.154 | -0.092 to 0.382 | 67 | 0.218 | 0.867 |
| Creatinine | DAR | -0.178 | -0.402 to 0.067 | 67 | 0.153 | 0.855 |
| Creatinine | DTABR | -0.181 | -0.405 to 0.064 | 67 | 0.146 | 0.841 |
| Creatinine | BSI | 0.031 | -0.213 to 0.271 | 67 | 0.804 | 0.956 |

## Table S2.2. Sensitivity analyses: Grid A repeated

Left: recordings with relative beta power >25% excluded (pharmacological beta proxy). Right: per-metric outlier trimming at median ± 3 × IQR, applied pairwise. Benjamini–Hochberg correction applied across all 156 tests within each analysis. No pair survived correction in either (smallest BH-adjusted p-value = 0.103 and 0.077 respectively).

| Marker | qEEG metric | Beta >25% excluded | | | Outlier-trimmed | | |
|---|---|---|---|---|---|---|---|
| | | ρ | n | p-value (BH) | ρ | n | p-value (BH) |
| CRP | Delta, absolute | -0.102 | 67 | 0.923 | -0.146 | 62 | 0.830 |
| CRP | Theta, absolute | -0.079 | 67 | 0.923 | -0.054 | 65 | 0.925 |
| CRP | Alpha, absolute | -0.055 | 67 | 0.923 | -0.041 | 68 | 0.927 |
| CRP | Beta, absolute | -0.045 | 67 | 0.923 | -0.013 | 67 | 0.982 |
| CRP | Total power | -0.098 | 67 | 0.923 | -0.134 | 61 | 0.830 |
| CRP | Delta, relative | -0.024 | 67 | 0.968 | -0.041 | 69 | 0.927 |
| CRP | Theta, relative | 0.046 | 67 | 0.923 | 0.052 | 69 | 0.925 |
| CRP | Alpha, relative | 0.058 | 67 | 0.923 | 0.062 | 68 | 0.921 |
| CRP | Beta, relative | -0.004 | 67 | 0.992 | 0.050 | 65 | 0.926 |
| CRP | DAR | -0.051 | 67 | 0.923 | -0.086 | 66 | 0.862 |
| CRP | DTABR | -0.005 | 67 | 0.992 | -0.024 | 67 | 0.954 |
| CRP | BSI | -0.071 | 67 | 0.923 | -0.068 | 69 | 0.910 |
| PCT | Delta, absolute | -0.102 | 57 | 0.923 | -0.099 | 52 | 0.862 |
| PCT | Theta, absolute | -0.222 | 57 | 0.854 | -0.214 | 56 | 0.756 |
| PCT | Alpha, absolute | -0.216 | 57 | 0.862 | -0.239 | 57 | 0.596 |
| PCT | Beta, absolute | -0.166 | 57 | 0.923 | -0.163 | 57 | 0.830 |
| PCT | Total power | -0.101 | 57 | 0.923 | -0.127 | 51 | 0.830 |
| PCT | Delta, relative | 0.107 | 57 | 0.923 | 0.102 | 58 | 0.862 |
| PCT | Theta, relative | -0.181 | 57 | 0.923 | -0.181 | 58 | 0.830 |
| PCT | Alpha, relative | -0.118 | 57 | 0.923 | -0.118 | 57 | 0.830 |
| PCT | Beta, relative | -0.037 | 57 | 0.958 | -0.035 | 56 | 0.954 |
| PCT | DAR | 0.106 | 57 | 0.923 | 0.043 | 55 | 0.937 |
| PCT | DTABR | 0.088 | 57 | 0.923 | 0.069 | 56 | 0.921 |
| PCT | BSI | -0.010 | 57 | 0.990 | -0.011 | 58 | 0.983 |
| Leucocytes | Delta, absolute | 0.035 | 65 | 0.958 | -0.039 | 61 | 0.940 |
| Leucocytes | Theta, absolute | 0.078 | 65 | 0.923 | 0.089 | 63 | 0.862 |
| Leucocytes | Alpha, absolute | 0.075 | 65 | 0.923 | 0.077 | 66 | 0.896 |
| Leucocytes | Beta, absolute | 0.106 | 65 | 0.923 | 0.089 | 65 | 0.862 |
| Leucocytes | Total power | 0.052 | 65 | 0.923 | -0.007 | 60 | 0.984 |
| Leucocytes | Delta, relative | -0.074 | 65 | 0.923 | -0.050 | 67 | 0.926 |
| Leucocytes | Theta, relative | 0.118 | 65 | 0.923 | 0.140 | 67 | 0.830 |
| Leucocytes | Alpha, relative | 0.106 | 65 | 0.923 | 0.092 | 66 | 0.862 |
| Leucocytes | Beta, relative | 0.101 | 65 | 0.923 | 0.098 | 63 | 0.862 |
| Leucocytes | DAR | -0.078 | 65 | 0.923 | -0.123 | 64 | 0.830 |
| Leucocytes | DTABR | -0.078 | 65 | 0.923 | -0.058 | 65 | 0.925 |

| | | | | | | | |
|---|---|---|---|---|---|---|---|
| Leucocytes | BSI | 0.094 | 65 | 0.923 | 0.084 | 67 | 0.862 |
| Neutrophils | Delta, absolute | -0.014 | 65 | 0.985 | -0.113 | 61 | 0.830 |
| Neutrophils | Theta, absolute | 0.055 | 65 | 0.923 | 0.054 | 63 | 0.925 |
| Neutrophils | Alpha, absolute | 0.066 | 65 | 0.923 | 0.037 | 66 | 0.940 |
| Neutrophils | Beta, absolute | 0.024 | 65 | 0.968 | 0.022 | 65 | 0.955 |
| Neutrophils | Total power | 0.003 | 65 | 0.992 | -0.119 | 60 | 0.830 |
| Neutrophils | Delta, relative | -0.071 | 65 | 0.923 | -0.067 | 67 | 0.910 |
| Neutrophils | Theta, relative | 0.112 | 65 | 0.923 | 0.132 | 67 | 0.830 |
| Neutrophils | Alpha, relative | 0.122 | 65 | 0.923 | 0.116 | 66 | 0.830 |
| Neutrophils | Beta, relative | 0.044 | 65 | 0.923 | 0.047 | 63 | 0.926 |
| Neutrophils | DAR | -0.101 | 65 | 0.923 | -0.178 | 64 | 0.830 |
| Neutrophils | DTABR | -0.050 | 65 | 0.923 | -0.072 | 65 | 0.910 |
| Neutrophils | BSI | 0.239 | 65 | 0.837 | 0.226 | 67 | 0.596 |
| Lymphocytes | Delta, absolute | 0.140 | 64 | 0.923 | 0.141 | 60 | 0.830 |
| Lymphocytes | Theta, absolute | 0.137 | 64 | 0.923 | 0.235 | 62 | 0.596 |
| Lymphocytes | Alpha, absolute | 0.053 | 64 | 0.923 | 0.079 | 65 | 0.896 |
| Lymphocytes | Beta, absolute | 0.097 | 64 | 0.923 | 0.028 | 64 | 0.954 |
| Lymphocytes | Total power | 0.150 | 64 | 0.923 | 0.198 | 59 | 0.784 |
| Lymphocytes | Delta, relative | 0.071 | 64 | 0.923 | 0.130 | 66 | 0.830 |
| Lymphocytes | Theta, relative | -0.011 | 64 | 0.990 | -0.007 | 66 | 0.984 |
| Lymphocytes | Alpha, relative | -0.107 | 64 | 0.923 | -0.121 | 65 | 0.830 |
| Lymphocytes | Beta, relative | -0.035 | 64 | 0.958 | -0.097 | 62 | 0.862 |
| Lymphocytes | DAR | 0.109 | 64 | 0.923 | 0.117 | 63 | 0.830 |
| Lymphocytes | DTABR | 0.058 | 64 | 0.923 | 0.122 | 64 | 0.830 |
| Lymphocytes | BSI | -0.153 | 64 | 0.923 | -0.155 | 66 | 0.830 |
| NLR | Delta, absolute | -0.058 | 64 | 0.923 | -0.111 | 60 | 0.848 |
| NLR | Theta, absolute | 0.031 | 64 | 0.963 | -0.037 | 62 | 0.940 |
| NLR | Alpha, absolute | 0.057 | 64 | 0.923 | 0.025 | 65 | 0.954 |
| NLR | Beta, absolute | -0.046 | 64 | 0.923 | 0.004 | 64 | 0.987 |
| NLR | Total power | -0.050 | 64 | 0.923 | -0.153 | 59 | 0.830 |
| NLR | Delta, relative | -0.045 | 64 | 0.923 | -0.099 | 66 | 0.862 |
| NLR | Theta, relative | 0.100 | 64 | 0.923 | 0.100 | 66 | 0.862 |
| NLR | Alpha, relative | 0.103 | 64 | 0.923 | 0.117 | 65 | 0.830 |
| NLR | Beta, relative | -0.034 | 64 | 0.958 | 0.022 | 62 | 0.955 |
| NLR | DAR | -0.096 | 64 | 0.923 | -0.152 | 63 | 0.830 |
| NLR | DTABR | 0.002 | 64 | 0.992 | -0.068 | 64 | 0.910 |
| NLR | BSI | 0.235 | 64 | 0.837 | 0.236 | 66 | 0.596 |
| PLR | Delta, absolute | -0.076 | 64 | 0.923 | -0.089 | 60 | 0.862 |
| PLR | Theta, absolute | 0.032 | 64 | 0.962 | -0.061 | 62 | 0.925 |
| PLR | Alpha, absolute | 0.027 | 64 | 0.963 | -0.002 | 65 | 0.992 |
| PLR | Beta, absolute | -0.101 | 64 | 0.923 | -0.049 | 64 | 0.926 |
| PLR | Total power | -0.078 | 64 | 0.923 | -0.141 | 59 | 0.830 |
| PLR | Delta, relative | -0.060 | 64 | 0.923 | -0.113 | 66 | 0.830 |
| PLR | Theta, relative | 0.111 | 64 | 0.923 | 0.111 | 66 | 0.830 |

| | | | | | | | |
|---|---|---|---|---|---|---|---|
| PLR | Alpha, relative | 0.050 | 64 | 0.923 | 0.057 | 65 | 0.925 |
| PLR | Beta, relative | -0.047 | 64 | 0.923 | -0.048 | 62 | 0.926 |
| PLR | DAR | -0.063 | 64 | 0.923 | -0.057 | 63 | 0.925 |
| PLR | DTABR | 0.008 | 64 | 0.992 | -0.045 | 64 | 0.927 |
| PLR | BSI | 0.313 | 64 | 0.331 | 0.311 | 66 | 0.345 |
| Platelets | Delta, absolute | -0.017 | 65 | 0.979 | -0.010 | 61 | 0.984 |
| Platelets | Theta, absolute | 0.077 | 65 | 0.923 | 0.069 | 63 | 0.910 |
| Platelets | Alpha, absolute | 0.028 | 65 | 0.963 | -0.021 | 66 | 0.955 |
| Platelets | Beta, absolute | -0.077 | 65 | 0.923 | -0.099 | 65 | 0.862 |
| Platelets | Total power | -0.023 | 65 | 0.968 | -0.050 | 60 | 0.926 |
| Platelets | Delta, relative | -0.050 | 65 | 0.923 | -0.020 | 67 | 0.956 |
| Platelets | Theta, relative | 0.084 | 65 | 0.923 | 0.086 | 67 | 0.862 |
| Platelets | Alpha, relative | 0.004 | 65 | 0.992 | -0.004 | 66 | 0.987 |
| Platelets | Beta, relative | -0.022 | 65 | 0.971 | -0.073 | 63 | 0.910 |
| Platelets | DAR | -0.011 | 65 | 0.990 | 0.056 | 64 | 0.925 |
| Platelets | DTABR | -0.001 | 65 | 0.993 | 0.027 | 65 | 0.954 |
| Platelets | BSI | 0.187 | 65 | 0.902 | 0.185 | 67 | 0.784 |
| Haemoglobin | Delta, absolute | 0.220 | 65 | 0.845 | 0.251 | 61 | 0.596 |
| Haemoglobin | Theta, absolute | 0.323 | 65 | 0.331 | 0.278 | 63 | 0.523 |
| Haemoglobin | Alpha, absolute | 0.288 | 65 | 0.412 | 0.225 | 66 | 0.596 |
| Haemoglobin | Beta, absolute | 0.205 | 65 | 0.854 | 0.150 | 65 | 0.830 |
| Haemoglobin | Total power | 0.225 | 65 | 0.845 | 0.217 | 60 | 0.724 |
| Haemoglobin | Delta, relative | -0.005 | 65 | 0.992 | 0.029 | 67 | 0.954 |
| Haemoglobin | Theta, relative | 0.041 | 65 | 0.939 | 0.012 | 67 | 0.982 |
| Haemoglobin | Alpha, relative | -0.082 | 65 | 0.923 | -0.081 | 66 | 0.884 |
| Haemoglobin | Beta, relative | -0.083 | 65 | 0.923 | -0.153 | 63 | 0.830 |
| Haemoglobin | DAR | 0.073 | 65 | 0.923 | 0.171 | 64 | 0.830 |
| Haemoglobin | DTABR | 0.075 | 65 | 0.923 | 0.116 | 65 | 0.830 |
| Haemoglobin | BSI | -0.152 | 65 | 0.923 | -0.132 | 67 | 0.830 |
| Albumin | Delta, absolute | 0.194 | 50 | 0.923 | 0.313 | 47 | 0.523 |
| Albumin | Theta, absolute | 0.266 | 50 | 0.837 | 0.344 | 50 | 0.345 |
| Albumin | Alpha, absolute | 0.199 | 50 | 0.923 | 0.143 | 51 | 0.830 |
| Albumin | Beta, absolute | 0.176 | 50 | 0.923 | 0.130 | 51 | 0.830 |
| Albumin | Total power | 0.211 | 50 | 0.909 | 0.320 | 46 | 0.523 |
| Albumin | Delta, relative | 0.017 | 50 | 0.985 | 0.080 | 52 | 0.910 |
| Albumin | Theta, relative | 0.012 | 50 | 0.990 | -0.030 | 52 | 0.954 |
| Albumin | Alpha, relative | -0.079 | 50 | 0.923 | -0.082 | 51 | 0.910 |
| Albumin | Beta, relative | -0.088 | 50 | 0.923 | -0.156 | 49 | 0.830 |
| Albumin | DAR | 0.107 | 50 | 0.923 | 0.166 | 50 | 0.830 |
| Albumin | DTABR | 0.097 | 50 | 0.923 | 0.102 | 51 | 0.862 |
| Albumin | BSI | 0.168 | 50 | 0.923 | 0.166 | 52 | 0.830 |
| AST | Delta, absolute | 0.157 | 65 | 0.923 | 0.120 | 61 | 0.830 |
| AST | Theta, absolute | -0.083 | 65 | 0.923 | -0.042 | 64 | 0.927 |
| AST | Alpha, absolute | -0.079 | 65 | 0.923 | -0.097 | 66 | 0.862 |
| AST | Beta, absolute | 0.155 | 65 | 0.923 | 0.117 | 65 | 0.830 |

| | | | | | | | |
|---|---|---|---|---|---|---|---|
| AST | Total power | 0.153 | 65 | 0.923 | 0.101 | 60 | 0.862 |
| AST | Delta, relative | 0.313 | 65 | 0.331 | 0.325 | 67 | 0.345 |
| AST | Theta, relative | -0.414 | 65 | 0.103 | -0.417 | 67 | 0.077 |
| AST | Alpha, relative | -0.326 | 65 | 0.331 | -0.322 | 66 | 0.345 |
| AST | Beta, relative | -0.105 | 65 | 0.923 | -0.149 | 63 | 0.830 |
| AST | DAR | 0.310 | 65 | 0.331 | 0.311 | 64 | 0.345 |
| AST | DTABR | 0.211 | 65 | 0.854 | 0.237 | 65 | 0.596 |
| AST | BSI | 0.190 | 65 | 0.902 | 0.184 | 67 | 0.784 |
| ALT | Delta, absolute | 0.046 | 65 | 0.923 | -0.008 | 61 | 0.984 |
| ALT | Theta, absolute | -0.134 | 65 | 0.923 | -0.142 | 64 | 0.830 |
| ALT | Alpha, absolute | -0.134 | 65 | 0.923 | -0.153 | 66 | 0.830 |
| ALT | Beta, absolute | 0.029 | 65 | 0.963 | -0.001 | 65 | 0.996 |
| ALT | Total power | 0.045 | 65 | 0.923 | -0.028 | 60 | 0.954 |
| ALT | Delta, relative | 0.195 | 65 | 0.872 | 0.166 | 67 | 0.830 |
| ALT | Theta, relative | -0.303 | 65 | 0.334 | -0.302 | 67 | 0.345 |
| ALT | Alpha, relative | -0.249 | 65 | 0.816 | -0.240 | 66 | 0.596 |
| ALT | Beta, relative | -0.069 | 65 | 0.923 | -0.149 | 63 | 0.830 |
| ALT | DAR | 0.212 | 65 | 0.854 | 0.145 | 64 | 0.830 |
| ALT | DTABR | 0.112 | 65 | 0.923 | 0.067 | 65 | 0.910 |
| ALT | BSI | 0.195 | 65 | 0.872 | 0.200 | 67 | 0.724 |
| Creatinine | Delta, absolute | -0.064 | 65 | 0.923 | 0.048 | 60 | 0.926 |
| Creatinine | Theta, absolute | -0.116 | 65 | 0.923 | -0.134 | 63 | 0.830 |
| Creatinine | Alpha, absolute | 0.040 | 65 | 0.939 | -0.017 | 66 | 0.970 |
| Creatinine | Beta, absolute | 0.020 | 65 | 0.973 | 0.054 | 65 | 0.925 |
| Creatinine | Total power | -0.074 | 65 | 0.923 | -0.027 | 59 | 0.954 |
| Creatinine | Delta, relative | -0.113 | 65 | 0.923 | -0.119 | 67 | 0.830 |
| Creatinine | Theta, relative | 0.019 | 65 | 0.979 | 0.014 | 67 | 0.979 |
| Creatinine | Alpha, relative | 0.221 | 65 | 0.845 | 0.223 | 66 | 0.596 |
| Creatinine | Beta, relative | 0.149 | 65 | 0.923 | 0.188 | 64 | 0.784 |
| Creatinine | DAR | -0.176 | 65 | 0.923 | -0.264 | 64 | 0.523 |
| Creatinine | DTABR | -0.177 | 65 | 0.923 | -0.206 | 65 | 0.724 |
| Creatinine | BSI | 0.027 | 65 | 0.963 | 0.031 | 67 | 0.954 |

Results for the third sensitivity analysis (montage-homogeneous 10–20 subset, n=52) are summarised above and reported in full in Table S2.3.

## Table S2.3. Grid A repeated in the montage-homogeneous standard 10–20 subset

Age-adjusted partial Spearman correlations restricted to children aged ≥8 months recorded with the standard international 10–20 montage (n=52; effective n per cell 36–50). Confidence intervals by Fisher z-transform with standard error $1/\sqrt{(n-4)}$. Benjamini–Hochberg correction applied across all 156 tests. No pair survived correction; the smallest BH-adjusted p-value was 0.869. Coefficients ranged from −0.370 to +0.355.

| Marker | qEEG metric | ρ | 95% CI | n | p-value (raw) | p-value (BH) |
|---|---|---|---|---|---|---|
| CRP | Delta, absolute | −0.160 | −0.423 to 0.126 | 50 | 0.271 | 0.906 |
| CRP | Theta, absolute | −0.119 | −0.387 to 0.168 | 50 | 0.415 | 0.906 |
| CRP | Alpha, absolute | −0.031 | −0.309 to 0.252 | 50 | 0.833 | 0.936 |
| CRP | Beta, absolute | 0.005 | −0.276 to 0.286 | 50 | 0.972 | 0.985 |
| CRP | Total power | −0.148 | −0.412 to 0.139 | 50 | 0.309 | 0.906 |
| CRP | Delta, relative | −0.105 | −0.375 to 0.182 | 50 | 0.473 | 0.906 |
| CRP | Theta, relative | 0.086 | −0.200 to 0.358 | 50 | 0.557 | 0.906 |
| CRP | Alpha, relative | 0.163 | −0.124 to 0.425 | 50 | 0.262 | 0.906 |
| CRP | Beta, relative | 0.106 | −0.181 to 0.376 | 50 | 0.469 | 0.906 |
| CRP | DAR | −0.142 | −0.407 to 0.145 | 50 | 0.331 | 0.906 |
| CRP | DTABR | −0.136 | −0.402 to 0.151 | 50 | 0.351 | 0.906 |
| CRP | BSI | −0.097 | −0.368 to 0.189 | 50 | 0.507 | 0.906 |
| PCT | Delta, absolute | −0.032 | −0.336 to 0.278 | 42 | 0.842 | 0.936 |
| PCT | Theta, absolute | −0.192 | −0.471 to 0.123 | 42 | 0.230 | 0.906 |
| PCT | Alpha, absolute | −0.098 | −0.394 to 0.216 | 42 | 0.541 | 0.906 |
| PCT | Beta, absolute | −0.019 | −0.324 to 0.291 | 42 | 0.908 | 0.950 |
| PCT | Total power | −0.031 | −0.336 to 0.279 | 42 | 0.845 | 0.936 |
| PCT | Delta, relative | 0.146 | −0.169 to 0.434 | 42 | 0.363 | 0.906 |
| PCT | Theta, relative | −0.229 | −0.502 to 0.084 | 42 | 0.149 | 0.897 |
| PCT | Alpha, relative | −0.092 | −0.389 to 0.222 | 42 | 0.567 | 0.912 |
| PCT | Beta, relative | −0.018 | −0.324 to 0.291 | 42 | 0.912 | 0.950 |
| PCT | DAR | 0.112 | −0.203 to | 42 | 0.487 | 0.906 |

| | | | | | | |
|---|---|---|---|---|---|---|
| | | | 0.405 | | | |
| PCT | DTABR | 0.068 | −0.245 to 0.368 | 42 | 0.674 | 0.935 |
| PCT | BSI | −0.233 | −0.505 to 0.080 | 42 | 0.142 | 0.897 |
| Leucocytes | Delta, absolute | −0.042 | −0.322 to 0.245 | 49 | 0.776 | 0.936 |
| Leucocytes | Theta, absolute | 0.111 | −0.179 to 0.383 | 49 | 0.452 | 0.906 |
| Leucocytes | Alpha, absolute | 0.122 | −0.168 to 0.392 | 49 | 0.409 | 0.906 |
| Leucocytes | Beta, absolute | 0.061 | −0.227 to 0.339 | 49 | 0.682 | 0.935 |
| Leucocytes | Total power | −0.014 | −0.297 to 0.271 | 49 | 0.926 | 0.950 |
| Leucocytes | Delta, relative | −0.161 | −0.425 to 0.129 | 49 | 0.276 | 0.906 |
| Leucocytes | Theta, relative | 0.237 | −0.051 to 0.488 | 49 | 0.105 | 0.897 |
| Leucocytes | Alpha, relative | 0.197 | −0.093 to 0.455 | 49 | 0.181 | 0.897 |
| Leucocytes | Beta, relative | 0.092 | −0.197 to 0.367 | 49 | 0.533 | 0.906 |
| Leucocytes | DAR | −0.177 | −0.439 to 0.112 | 49 | 0.228 | 0.906 |
| Leucocytes | DTABR | −0.113 | −0.384 to 0.177 | 49 | 0.446 | 0.906 |
| Leucocytes | BSI | 0.104 | −0.186 to 0.377 | 49 | 0.482 | 0.906 |
| Neutrophils | Delta, absolute | −0.093 | −0.368 to 0.196 | 49 | 0.529 | 0.906 |
| Neutrophils | Theta, absolute | 0.022 | −0.264 to 0.304 | 49 | 0.883 | 0.950 |
| Neutrophils | Alpha, absolute | 0.081 | −0.208 to 0.357 | 49 | 0.586 | 0.923 |
| Neutrophils | Beta, absolute | −0.045 | −0.325 to 0.242 | 49 | 0.759 | 0.936 |
| Neutrophils | Total power | −0.063 | −0.341 to 0.225 | 49 | 0.669 | 0.935 |
| Neutrophils | Delta, relative | −0.096 | −0.370 to 0.193 | 49 | 0.517 | 0.906 |
| Neutrophils | Theta, relative | 0.136 | −0.155 to 0.404 | 49 | 0.358 | 0.906 |
| Neutrophils | Alpha, relative | 0.194 | −0.096 to 0.453 | 49 | 0.187 | 0.897 |

| | | | | | | |
|---|---|---|---|---|---|---|
| Neutrophils | Beta, relative | 0.033 | −0.253 to 0.314 | 49 | 0.824 | 0.936 |
| Neutrophils | DAR | −0.164 | −0.428 to 0.126 | 49 | 0.265 | 0.906 |
| Neutrophils | DTABR | −0.068 | −0.345 to 0.221 | 49 | 0.648 | 0.935 |
| Neutrophils | BSI | 0.198 | −0.092 to 0.456 | 49 | 0.178 | 0.897 |
| Lymphocytes | Delta, absolute | 0.144 | −0.149 to 0.414 | 48 | 0.334 | 0.906 |
| Lymphocytes | Theta, absolute | 0.248 | −0.042 to 0.499 | 48 | 0.093 | 0.897 |
| Lymphocytes | Alpha, absolute | 0.078 | −0.214 to 0.357 | 48 | 0.603 | 0.923 |
| Lymphocytes | Beta, absolute | 0.037 | −0.253 to 0.321 | 48 | 0.804 | 0.936 |
| Lymphocytes | Total power | 0.156 | −0.137 to 0.424 | 48 | 0.296 | 0.906 |
| Lymphocytes | Delta, relative | 0.041 | −0.249 to 0.324 | 48 | 0.784 | 0.936 |
| Lymphocytes | Theta, relative | 0.116 | −0.177 to 0.390 | 48 | 0.437 | 0.906 |
| Lymphocytes | Alpha, relative | −0.095 | −0.372 to 0.198 | 48 | 0.526 | 0.906 |
| Lymphocytes | Beta, relative | −0.092 | −0.370 to 0.200 | 48 | 0.537 | 0.906 |
| Lymphocytes | DAR | 0.074 | −0.218 to 0.354 | 48 | 0.620 | 0.930 |
| Lymphocytes | DTABR | 0.084 | −0.208 to 0.362 | 48 | 0.576 | 0.916 |
| Lymphocytes | BSI | −0.079 | −0.358 to 0.213 | 48 | 0.597 | 0.923 |
| NLR | Delta, absolute | −0.155 | −0.424 to 0.138 | 48 | 0.297 | 0.906 |
| NLR | Theta, absolute | −0.089 | −0.367 to 0.203 | 48 | 0.552 | 0.906 |
| NLR | Alpha, absolute | 0.037 | −0.253 to 0.321 | 48 | 0.805 | 0.936 |
| NLR | Beta, absolute | −0.061 | −0.342 to 0.230 | 48 | 0.683 | 0.935 |
| NLR | Total power | −0.133 | −0.405 to 0.160 | 48 | 0.373 | 0.906 |
| NLR | Delta, relative | −0.118 | −0.392 to 0.176 | 48 | 0.431 | 0.906 |
| NLR | Theta, | 0.063 | −0.228 to | 48 | 0.673 | 0.935 |

| | | | | | | |
|---|---|---|---|---|---|---|
| | relative | | 0.344 | | | |
| NLR | Alpha, relative | 0.200 | −0.092 to 0.461 | 48 | 0.177 | 0.897 |
| NLR | Beta, relative | 0.075 | −0.217 to 0.354 | 48 | 0.619 | 0.930 |
| NLR | DAR | −0.175 | −0.440 to 0.119 | 48 | 0.241 | 0.906 |
| NLR | DTABR | −0.097 | −0.374 to 0.195 | 48 | 0.515 | 0.906 |
| NLR | BSI | 0.195 | −0.098 to 0.456 | 48 | 0.190 | 0.897 |
| PLR | Delta, absolute | −0.121 | −0.395 to 0.172 | 48 | 0.416 | 0.906 |
| PLR | Theta, absolute | −0.126 | −0.398 to 0.168 | 48 | 0.400 | 0.906 |
| PLR | Alpha, absolute | −0.096 | −0.373 to 0.197 | 48 | 0.521 | 0.906 |
| PLR | Beta, absolute | −0.213 | −0.472 to 0.079 | 48 | 0.150 | 0.897 |
| PLR | Total power | −0.135 | −0.406 to 0.159 | 48 | 0.367 | 0.906 |
| PLR | Delta, relative | −0.019 | −0.304 to 0.270 | 48 | 0.900 | 0.950 |
| PLR | Theta, relative | 0.001 | −0.286 to 0.288 | 48 | 0.992 | 0.996 |
| PLR | Alpha, relative | 0.048 | −0.243 to 0.330 | 48 | 0.750 | 0.936 |
| PLR | Beta, relative | −0.035 | −0.319 to 0.255 | 48 | 0.818 | 0.936 |
| PLR | DAR | −0.039 | −0.322 to 0.251 | 48 | 0.796 | 0.936 |
| PLR | DTABR | 0.021 | −0.268 to 0.306 | 48 | 0.890 | 0.950 |
| PLR | BSI | 0.347 | 0.067 to 0.577 | 48 | 0.017 | 0.869 |
| Platelets | Delta, absolute | −0.014 | −0.297 to 0.271 | 49 | 0.923 | 0.950 |
| Platelets | Theta, absolute | 0.016 | −0.270 to 0.298 | 49 | 0.916 | 0.950 |
| Platelets | Alpha, absolute | −0.099 | −0.372 to 0.191 | 49 | 0.504 | 0.906 |
| Platelets | Beta, absolute | −0.271 | −0.516 to 0.014 | 49 | 0.062 | 0.897 |
| Platelets | Total power | −0.041 | −0.322 to 0.246 | 49 | 0.780 | 0.936 |

| | | | | | | |
|---|---|---|---|---|---|---|
| Platelets | Delta, relative | 0.030 | −0.256 to 0.311 | 49 | 0.840 | 0.936 |
| Platelets | Theta, relative | 0.065 | −0.223 to 0.343 | 49 | 0.662 | 0.935 |
| Platelets | Alpha, relative | −0.037 | −0.318 to 0.250 | 49 | 0.801 | 0.936 |
| Platelets | Beta, relative | −0.108 | −0.381 to 0.181 | 49 | 0.463 | 0.906 |
| Platelets | DAR | 0.037 | −0.250 to 0.318 | 49 | 0.802 | 0.936 |
| Platelets | DTABR | 0.106 | −0.184 to 0.379 | 49 | 0.473 | 0.906 |
| Platelets | BSI | 0.260 | −0.026 to 0.507 | 49 | 0.074 | 0.897 |
| Haemoglobin | Delta, absolute | 0.269 | −0.016 to 0.514 | 49 | 0.064 | 0.897 |
| Haemoglobin | Theta, absolute | 0.320 | 0.039 to 0.553 | 49 | 0.027 | 0.897 |
| Haemoglobin | Alpha, absolute | 0.149 | −0.141 to 0.416 | 49 | 0.312 | 0.906 |
| Haemoglobin | Beta, absolute | 0.070 | −0.219 to 0.347 | 49 | 0.637 | 0.935 |
| Haemoglobin | Total power | 0.274 | −0.011 to 0.518 | 49 | 0.059 | 0.897 |
| Haemoglobin | Delta, relative | 0.118 | −0.172 to 0.389 | 49 | 0.424 | 0.906 |
| Haemoglobin | Theta, relative | −0.049 | −0.328 to 0.239 | 49 | 0.742 | 0.936 |
| Haemoglobin | Alpha, relative | −0.230 | −0.482 to 0.058 | 49 | 0.116 | 0.897 |
| Haemoglobin | Beta, relative | −0.178 | −0.440 to 0.112 | 49 | 0.226 | 0.906 |
| Haemoglobin | DAR | 0.207 | −0.081 to 0.464 | 49 | 0.157 | 0.897 |
| Haemoglobin | DTABR | 0.231 | −0.057 to 0.483 | 49 | 0.114 | 0.897 |
| Haemoglobin | BSI | 0.037 | −0.250 to 0.318 | 49 | 0.802 | 0.936 |
| Albumin | Delta, absolute | 0.227 | −0.115 to 0.521 | 36 | 0.189 | 0.897 |
| Albumin | Theta, absolute | 0.287 | −0.051 to 0.566 | 36 | 0.094 | 0.897 |
| Albumin | Alpha, absolute | 0.060 | −0.279 to 0.385 | 36 | 0.734 | 0.936 |
| Albumin | Beta, | 0.024 | −0.312 to | 36 | 0.890 | 0.950 |

| | | | | | | |
|---|---|---|---|---|---|---|
| | absolute | | 0.355 | | | |
| Albumin | Total power | 0.235 | −0.106 to 0.527 | 36 | 0.174 | 0.897 |
| Albumin | Delta, relative | 0.111 | −0.231 to 0.429 | 36 | 0.525 | 0.906 |
| Albumin | Theta, relative | −0.034 | −0.363 to 0.303 | 36 | 0.846 | 0.936 |
| Albumin | Alpha, relative | −0.143 | −0.455 to 0.200 | 36 | 0.412 | 0.906 |
| Albumin | Beta, relative | −0.157 | −0.465 to 0.186 | 36 | 0.369 | 0.906 |
| Albumin | DAR | 0.199 | −0.143 to 0.499 | 36 | 0.251 | 0.906 |
| Albumin | DTABR | 0.193 | −0.149 to 0.495 | 36 | 0.266 | 0.906 |
| Albumin | BSI | 0.240 | −0.101 to 0.531 | 36 | 0.165 | 0.897 |
| AST | Delta, absolute | 0.200 | −0.093 to 0.461 | 48 | 0.178 | 0.897 |
| AST | Theta, absolute | 0.001 | −0.286 to 0.288 | 48 | 0.996 | 0.996 |
| AST | Alpha, absolute | 0.122 | −0.171 to 0.395 | 48 | 0.415 | 0.906 |
| AST | Beta, absolute | 0.355 | 0.076 to 0.583 | 48 | 0.014 | 0.869 |
| AST | Total power | 0.204 | −0.088 to 0.464 | 48 | 0.169 | 0.897 |
| AST | Delta, relative | 0.218 | −0.074 to 0.475 | 48 | 0.141 | 0.897 |
| AST | Theta, relative | −0.370 | −0.594 to −0.092 | 48 | 0.011 | 0.869 |
| AST | Alpha, relative | −0.190 | −0.453 to 0.103 | 48 | 0.200 | 0.906 |
| AST | Beta, relative | 0.034 | −0.255 to 0.318 | 48 | 0.819 | 0.936 |
| AST | DAR | 0.199 | −0.094 to 0.460 | 48 | 0.180 | 0.897 |
| AST | DTABR | 0.088 | −0.204 to 0.366 | 48 | 0.555 | 0.906 |
| AST | BSI | 0.122 | −0.171 to 0.395 | 48 | 0.413 | 0.906 |
| ALT | Delta, absolute | 0.036 | −0.254 to 0.320 | 48 | 0.812 | 0.936 |
| ALT | Theta, absolute | −0.117 | −0.391 to 0.176 | 48 | 0.433 | 0.906 |

| | | | | | | |
|---|---|---|---|---|---|---|
| ALT | Alpha, absolute | −0.027 | −0.311 to 0.263 | 48 | 0.859 | 0.944 |
| ALT | Beta, absolute | 0.155 | −0.139 to 0.423 | 48 | 0.299 | 0.906 |
| ALT | Total power | 0.051 | −0.240 to 0.333 | 48 | 0.734 | 0.936 |
| ALT | Delta, relative | 0.135 | −0.158 to 0.406 | 48 | 0.365 | 0.906 |
| ALT | Theta, relative | −0.314 | −0.552 to −0.030 | 48 | 0.031 | 0.897 |
| ALT | Alpha, relative | −0.167 | −0.433 to 0.126 | 48 | 0.262 | 0.906 |
| ALT | Beta, relative | 0.015 | −0.273 to 0.301 | 48 | 0.918 | 0.950 |
| ALT | DAR | 0.153 | −0.140 to 0.422 | 48 | 0.305 | 0.906 |
| ALT | DTABR | 0.045 | −0.246 to 0.328 | 48 | 0.766 | 0.936 |
| ALT | BSI | 0.224 | −0.067 to 0.480 | 48 | 0.130 | 0.897 |
| Creatinine | Delta, absolute | −0.066 | −0.341 to 0.220 | 50 | 0.654 | 0.935 |
| Creatinine | Theta, absolute | −0.254 | −0.500 to 0.029 | 50 | 0.078 | 0.897 |
| Creatinine | Alpha, absolute | −0.209 | −0.463 to 0.077 | 50 | 0.150 | 0.897 |
| Creatinine | Beta, absolute | −0.127 | −0.394 to 0.160 | 50 | 0.384 | 0.906 |
| Creatinine | Total power | −0.097 | −0.369 to 0.189 | 50 | 0.505 | 0.906 |
| Creatinine | Delta, relative | 0.038 | −0.246 to 0.316 | 50 | 0.797 | 0.936 |
| Creatinine | Theta, relative | −0.126 | −0.394 to 0.161 | 50 | 0.387 | 0.906 |
| Creatinine | Alpha, relative | 0.064 | −0.221 to 0.339 | 50 | 0.663 | 0.935 |
| Creatinine | Beta, relative | 0.078 | −0.208 to 0.351 | 50 | 0.594 | 0.923 |
| Creatinine | DAR | −0.007 | −0.288 to 0.275 | 50 | 0.962 | 0.981 |
| Creatinine | DTABR | −0.057 | −0.333 to 0.228 | 50 | 0.698 | 0.936 |
| Creatinine | BSI | 0.055 | −0.230 to 0.331 | 50 | 0.706 | 0.936 |

## Table S2.4. Aetiological composition and inflammatory markers in children with cortical silence (Synek 5) and preserved cortical activity (Synek 1–4), combined cohort (n=97)

| Aetiology | Total, n | Cortical silence, n (%) | PCT, ng/ml - silence | PCT, ng/ml - preserved activity | CRP, mg/dl - silence | CRP, mg/dl - preserved activity |
|---|---|---|---|---|---|---|
| Hypoxic–ischaemic | 25 | 12 (48) | 13.82 (0.79–47.40) [11] | 0.68 (0.20–1.90) [11] | 12.30 (4.88–18.18) [11] | 0.41 (0.30–1.15) [13] |
| Inflammatory | 39 | 6 (15) | 2.86 (0.91–6.86) [5] | 0.30 (0.14–2.15) [30] | 16.39 (4.84–19.44) [5] | 0.53 (0.25–6.43) [32] |
| Trauma | 8 | 6 (75) | 3.22 (2.20–6.23) [6] | 0.23 [1] | 13.66 (6.88–18.27) [6] | 0.41 and 2.61 [2] |
| Epilepsy | 25 | 2 (8) | 0.16 and 0.17 [2] | 0.14 (0.10–0.37) [16] | 0.39 and 2.03 [2] | 1.13 (0.28–3.00) [22] |

Values are median (IQR) [available n]; individual values are shown where n≤2. Hypoxic–ischaemic, post-cardiac arrest and post-hypoxic encephalopathy; inflammatory, sepsis and meningoencephalitis. Descriptive data; no statistical tests were performed because several cells are small. Marker n may be lower than group n because of missing values.

## Table S2.5. Statistical test by type of comparison.

| Comparison | Test |
|---|---|
| Continuous variables, two groups (spectral vs cortical-silence cohort) | Mann–Whitney U test (normal approximation) |
| Proportions, two groups | Fisher's exact test |
| Continuous variables, more than two groups (Synek grades 1–4; diagnostic groups) | Kruskal–Wallis test with tie correction |
| Association with Synek grade or between continuous variables | Spearman correlation |
| Age-adjusted associations | Partial Spearman correlation by rank residualisation (Section S2.3) |
| Non-monotonic pattern | Quadratic term in linear regression |

| | |
|---|---|
| across grades 1–4 (Grid B) | |
| Cortical silence vs preserved activity, with adjustment | Logistic regression on ln(marker + 0.01) (Section S2.6) |
| In-hospital death | Univariable logistic regression |
| Inter-rater agreement on Synek grade | Cohen's κ, unweighted and quadratic-weighted |
| Multiple testing | Benjamini–Hochberg within each family (Section S2.1) |
